# A Comprehensive Review on Advanced Sustainable Woven Natural Fibre Polymer Composites

**DOI:** 10.3390/polym13030471

**Published:** 2021-02-02

**Authors:** H. A. Aisyah, M. T. Paridah, S. M. Sapuan, R. A. Ilyas, A. Khalina, N. M. Nurazzi, S. H. Lee, C. H. Lee

**Affiliations:** 1 Institute of Tropical Forestry and Forest Products (INTROP), Universiti Putra Malaysia (UPM), Serdang 43400, Malaysia; parida.introp@gmail.com (M.T.P.); lee_seng@upm.edu.my (S.H.L.); leechinghao@upm.edu.my (C.H.L.); 2 Faculty of Engineering, Universiti Putra Malaysia (UPM), Serdang 43400, Malaysia; 3 Sustainable Waste Management Research Group (SWAM), School of Chemical and Energy Engineering, Faculty of Engineering, Universiti Teknologi Malaysia (UTM), Johor Bahru 81310, Malaysia; 4 Centre for Advanced Composite Materials, Universiti Teknologi Malaysia (UTM), Johor Bahru 81310, Malaysia; 5 Center for Defence Foundation Studies, National Defence University of Malaysia, Kem Sungai Besi, Kuala Lumpur 57000, Malaysia; mohd.nurazzi@gmail.com

**Keywords:** natural fibre, yarn, fabric, weave, woven composite, strength

## Abstract

Over the last decade, the progressive application of natural fibres in polymer composites has had a major effect in alleviating environmental impacts. Recently, there is a growing interest in the development of green materials in a woven form by utilising natural fibres from lignocellulosic materials for many applications such as structural, non-structural composites, household utilities, automobile parts, aerospace components, flooring, and ballistic materials. Woven materials are one of the most promising materials for substituting or hybridising with synthetic polymeric materials in the production of natural fibre polymer composites (NFPCs). These woven materials are flexible, able to be tailored to the specific needs and have better mechanical properties due to their weaving structures. Seeing that the potential advantages of woven materials in the fabrication of NFPC, this paper presents a detailed review of studies related to woven materials. A variety of factors that influence the properties of the resultant woven NFRC such as yarn characteristics, fabric properties as well as manufacturing parameters were discussed. Past and current research efforts on the development of woven NFPCs from various polymer matrices including polypropylene, polylactic acid, epoxy and polyester and the properties of the resultant composites were also compiled. Last but not least, the applications, challenges, and prospects in the field also were highlighted.

## 1. Introduction

Composites are materials that consist of two or more physical and chemical distinct phases, separated by an interface [1,2]. Such different phases are combined in order to yield a composites with stronger structural or functional properties that the individual components [3]. Composite materials benefit over non-composite materials for possessing low weight, corrosion resistance, high fatigue power, and quick installation [4,5]. Composites are commonly used in the manufacture of aircraft frames, electronic devices and packaging, transmission towers, medical equipment, space vehicles, and house construction [6,7,8]. There are three types of composites: ceramic matrix composites (CMCs), polymer matrix composites (PMCs), and metal matrix composites (MMCs). Composites are classified into particulate, fibrous, and laminate composites according to their types of reinforcement. Particulate composites are composed of particles, fibrous composites are made up of fibres and laminate composites are laminated. Fibrous composites are made of natural or synthetic fibre, where natural fibre composites are known as biofibre composites or biocomposites. Such natural fibres are also classified as non-biodegradable matrix and biodegradable matrix [9]. Natural fibre and biodegradable polymers produce composites known as biobased green composites that are further grouped into hybrid and textile composites [10,11,12,13,14]. Hybrid composites are a mixture of two or more types of fibres [15].

Most commercially manufactured composites use a polymer matrix material often referred to as a resin solution. PMC is low cost and easy to manufacture, making it very popular. The use of non-reinforced polymers as structural materials is restrained by their poor mechanical properties, i.e., strength, modulus, and impact resistance. A fibre-reinforced polymer composite (FRPC) is a PMC reinforced by polymers produced from strong fibrous networks. FRPC is known for certain characteristics such as high specific strength and toughness, high resistance to fractures, good abrasive strength, great impact resistance, excellent corrosion resistance, and being relatively cheap [16]. Glass, carbon, basalt, Kevlar, and boron fibres are used for reinforcement in FRPC and are widely recognised as structural and non-structural applications materials [3,17]. However, their non-biodegradability could contribute to serious environmental problems.

Currently, there is a growing interest of using natural fibres in PMC production in many applications, such as structural applications [18,19,20], construction [21], parts for automobiles and aerospace [22,23], fire extinguishers [24], furniture [25], packaging [26], ballistic laminate composites applications [27,28,29], and biomedical applications [30,31,32]. Natural fibres from plants such as jute, kenaf, bamboo, coir, sisal, and pineapple possess very high resistance and are suitable for many load-bearing applications. Furthermore, natural fibres are preferred over synthetic fibres because they are abundant, renewable and biodegradable, features which synthetic composites cannot offer. Natural fibres can be used as green substitutes due to their advantages such as light weight, good mechanical properties and low density with good strength.

## 2. Natural Fibres

Natural fibres come from three main sources: plants, animals and minerals. Due to its their easy availability, degradability, renewability, and environmental friendliness plant fibres have gained interest from scientists, researchers, and engineers [33,34,35]. Natural fibre-based composite materials have broad applications in industrial components, automotive sector, building structures, furniture and packaging that increase the environmental sustainability by contributing to the production of sustainable materials as alternatives to synthetic or man-made fibres [36]. Biomass from agricultural waste such as oil palm fronds [37], *Tamarindus indica* nut [38], empty fruit bunch (EFB) [39], coir [40], straws, husks [41], sugar palm [42,43,44,45,46,47], water hyacinth [12], and sugarcane bagasse [48,49] are classified as natural plant fibres. This group also includes some fibre crops, such as cotton, ramie, flax, bamboo, kenaf, jute, abaca, sisal, and hemp. Plant fibres are lignocellulosic materials consisting mainly of cellulose, hemicellulose and lignin [50]. These fibres are commonly used as reinforcements in the FRPC due to their low density, good mechanical properties, recyclable, and high strength. Table 1 shows the mechanical properties of some plant fibres that are widely used in the FRPC industry.

Even though natural fibre reinforcement has been proven to provide strength enhancement to polymer composites, the natural fibres are still not as ‘favourable’ as synthetic fibres. This is because of the high batch-to-batch variation of their properties and even the fact that every single natural fibre behaves slightly different from others [32]. To understand this reinforcing gap, several drawbacks of using natural fibres must be identified. According to Chattopadhyay et al. [62], the properties of plant fibre depend mainly on the nature, habitat, age, and method of plant extraction. The strength of the natural fibre-reinforced polymer composites relies primarily on the fibres’ cellulose component and fibre/matrix interfacial bonding conditions [63]. Natural fibres are grown from the soil, in different geographical regions, climates, and time-to-harvest that produce natural fibres with a variety in physical and chemical structures, and thereby different performances for every single fibre [64]. However, many fibre retting processes are available to produce fibres from their bundles, depending on geographical, labour skills, and cost-effective factors. Hence, retted natural fibres with different chemical and physical conditions are obtained. Besides, plant variety also yields changes in composites’ performance [65]. On the other hand, irregularly shaped fibres might become debonded at lower loads, starting to split from each other, with fibrillation occurring [66]. The inconsistent diameter of fibres was reported to be impactful on mechanical properties [67].

In addition, the hydrophobicity of the plant fibres is exhibited by their high absorbance characteristics, where the presence of a large number of hydroxyl groups in them allows water to be absorbed into the fibres, altering the composite’s mechanical properties. Plant fibres also display weak adhesion bonding between polymer matrixes and low thermal stability that most plant fibres tend to degrade at high temperatures. Besides, plant fibres also have limited mechanical strength, particularly tensile strength, compared to synthetic fibres, which results in undesirable composite performance [68]. Even though treatments could assist in homogenising the uniformity of the natural fibres and thereby improving interface adhesion, natural fibre reinforcement in composites is highly inconsistent as a result of the abovementioned drawbacks of natural fibres [69].

### 2.1. Natural Fibre Polymer Composite (NFPC)

Natural fibre polymer composite (NFPC) is a composite material consisting of a polymer matrix mixed with natural fibres from banana, oil palm, jute, flax, kenaf or ramie. There are two types of common polymers: thermoplastics, and thermosets. Thermoplastic polymers consist of one-or two-dimensional molecular structures, which are flexible when subjected to heat or force, and reshape under applied pressure and heat. The popular types of thermoplastics commonly used for NFPC are polyethylene (PE), polypropylene (PP) and polyvinyl chloride (PVC). Thermoset polymers, on the other hand, consist of polymers that cross-link together and create an irreversible chemical bond upon application of pressure and heat. This feature of thermoset polymers makes them suitable for high-temperature applications, such as electronics parts in electrical appliances. Other excellent properties of thermoset polymers include high modulus and high strength properties [70]. Phenolic, polyester (PE) and epoxy resins are the most widely used thermoset polymers.

### 2.2. Woven Natural Fibre Reinforced Polymer Composite

Natural fibres are available in a wide range of forms in fibre-reinforced polymer composites, such as continuous, randomised, and fabric [71,72]. Fabric is made by the textile processes of braiding, knitting, stitching, or weaving. Of these processes, weaving is one of the most commonly employed in fabrics for composite manufacturing because of the high productivity combined with the flexibility to produce a diverse range of fabric structures. Woven composites are commonly known as textile composites, made up of technical textiles rather than the conventional textiles. In addition, the use of a weaving techniques to produce fabrics has received much attention because they provide composites with superior mechanical properties than those obtained using both knitting and braiding techniques. Moreover, woven composites also have the ability to produce near net shape preforms or fabrics with high flexibility and stability. Weaving involves a warp that interlaces a set of longer threads and a weft that crosses a set of threads which is done by using a loom. However, some weavings are still done by hand despite the fact that they are typically merchandised. The main advantage of the use of weaved fabrics is the possibility of pre-directing the filaments in the intended direction. Table 2 summarises the studies on woven fibre reinforced polymer composites with natural fibres as reinforcement.

## 3. Factors Influencing Woven Polymer Composites Properties

Woven polymer composites have excellent mechanical strengths, i.e., rigidity, strength, and dimensional stability compared to unidirectional fibre composites for a number of applications in fibre reinforced polymers. The development of appropriate reinforcement is crucial to the achievement of optimum mechanical properties, in particular in the manufacturing of hybrid composites using natural fibre, in any forms, with a view to the manipulation of final composite properties. In the case of hybrid woven composites, textile engineering concepts are used. This can be accomplished by using the correct yarn size, where the correct choice of yarn size is capable to provide optimum force value and strength to withstand the deformation of the woven fabric and the good mechanical properties of the composites [77]. In addition to the properties of matrix and yarn, the strength and hardness of woven fabric strengthened composites are determined by the structural parameters of materials, i.e., the fabrics counts and weave designs.

### 3.1. Yarn Properties

**Figure 1:**
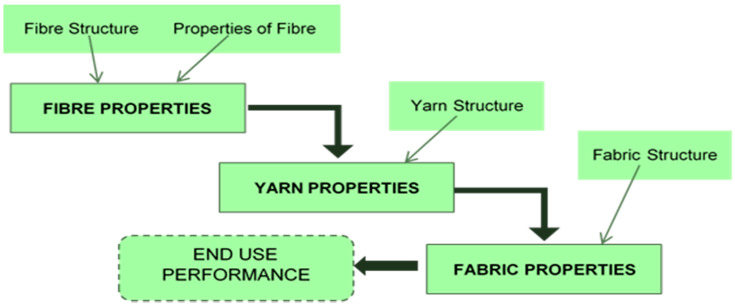
The connection between fibre, yarn, and fabric properties in textile composite point of view.

Yarn is a consistent strand used in textile weaving and knitting, made of natural or man-made fibre or filaments. The Textile Institute describes yarn as a product of substantial length and a comparatively small cross-section composed of fibres with or without twisting. The basic unit of the raw material to produce yarn is the textile fibre. Figure 1 illustrates the inter-relationships between the structure and properties of fibres, yarns, and fabrics. The fibre-to-yarn conversion process is influenced by a variety of factors, including raw material properties, processing method, machinery and machine operation conditions. For successful conversions into yarns and fabrics, the textile fibre must possess sufficient length, flexibility, fineness, and strength. A fibre of an incredible length is referred to as a filament. A yarn, consisting of either staple or filament fibre or other comparable forms and compounds come in different sizes and shapes, is relatively strong, and flexible.

**Figure 2:**
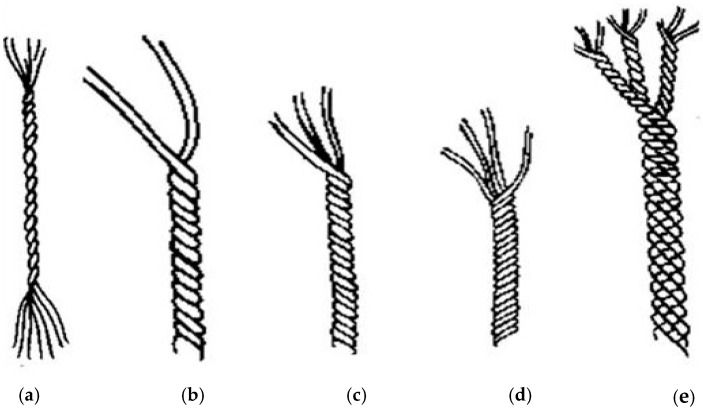
A sketch of different types of yarn. (**a**) simple ply yarn, (**b**) double-ply yarn, (**c**) three-ply yarn, (**d**) four-ply yarn and, (**e**) simple cord yarn. Source: Figure reproduced with copyright permission from Mobarak Hossain et al. [96]. This article is an open-access article distributed under the terms and conditions of the Creative Commons Attribution (CC BY) license (http://creativecommons.org/licenses/by/4.0/).

The easiest continuous fibre-material strand is a single yarn. If that strand comprises staple fibre, it is a staple yarn or spun yarn. If the strand is a single continuous filament, it is called a monofilament yarn, or just a monofilament. A strand is described as a multi yarn or a simple multifilament if the strand encompasses a small bundle of single filaments. A flat ribbon, fragmented from a thin film, is known as the tape yarn. A composite of filaments and staples could also be manufactured out of a single strand, with the filaments maintaining in the centre, wrapped around by staple fibre. Such a yarn is referred to as a core yarn, a type of composite yarn. Plied yarn is also referred to as a folded yarn or twisted yarn that generate a two-ply or two-fold yarn if two single yarns are twisted together. It forms a multi-ply yarn if there are more than two single yarns involved as shown in Figure 2.

Various kinds of plant fibre, such as cotton, jute, flax, and hemp fibre can be processed into yarns. Most of the plant fibres are located in bundles on the periphery of the stem just below the epidermis. When the plants have reached maturity, they are ready to harvest. After harvesting, the fibres are separated from the plant stems by two processes: retting and mechanical extraction to eliminate the tissue that interconnects the single fibre, i.e., the middle lamella. After retting, the fibres are removed from the stems via a mechanical process. Various extraction techniques can be used with the principle that the origin of the stems can be broken down into shorter lengths [97]. The extracted fibre content is only partly divided into a single fibre and an additional step is essential to increase the separation of the fibre, which is defined as defibration. Defibration is accomplished by carding in yarn manufacturing, where the fibre bundles are divided by toothed surfaces acting against each other in this process [98]. The set of fibre after defibration is referred to as filament, which corresponds to a sliver in textile terminology.

To ensure less yarn properties variation, the arrangement of the yarn must be sorted before twisting to achieve a high degree of yarn regularity. This is performed by two processes: (i) combing to align the fibre and eliminate the shortest portion of the fibre; and (ii) drafting to straighten the fibre and verify that the number of fibres in the cross-section of the filament is within the specified constraints [98,99]. In combing, the filament progresses through a series of pinned rollers that combs the short and twisted fibre and aligns the long fibres. In the drafting process, the filament is often passed through a series of rollers. As a result, when applied to a twisted filament, the fibre slips apart at low-fibre friction locations, which are similar to low-twist locations, i.e., dense locations. Thus, the drafting primarily affects the thick locations of the filament before they exceed the volume of the thin locations. After that, the twists are redistributed and the whole filament is uniformly affected by the drafting [98].

**Figure 3:**
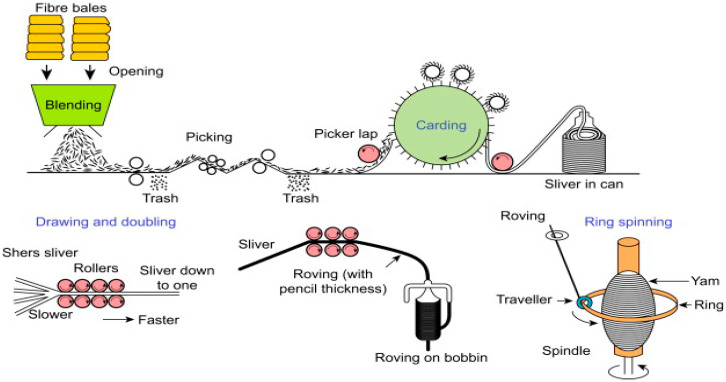
Yarn processing flow chart of the conventional ring-spinning process. Figure reproduced from Alagirusamy and Das [101] with copyright permission from Elsevier.

Parallelised fibre filaments are supplied from the drafting rollers and the twists are applied by the arrangement of: (i) the traveller rotating freely on the ring, (ii) the ring distributing the yarn on the coil, and (iii) the bobbin which is rotating as shown in Figure 3. Each rotation of the traveller embeds one twist, and the number of turns (i.e., turns) per length is managed by varying the delivery speed of the drafting rollers and the bobbin rotation speed [100]. The twisting operation forces the fibre to form a helical structure. Owing to the inherent sliding friction between the fibres, the fibres are slightly stretched and the strain is applied to the inside of the yarn. This increases the frictional forces between the fibre and the axial resistance of the yarn [98]. Therefore, when deciding the breaking load of yarn, the twist number is the key factor, i.e., with the twist number, the breaking load is increased to a maximum value, where it begins to decrease.

Given its effect on fibre production and product efficiency, yarn linear density is one of the essential parameters of fibre quality. A yarn numbering system which is determined by weight per unit length of yarn is linear density. In Tex, Decitex, and Denier, the main unit framework is normally present. The thinner the yarn, the lower the linear density in the linear density method. The unit referring to the weight of a yarn in gram for a length of 1000 m (constant) is used in a Tex system. The linear density of the yarn or the fabric can be viewed as a measure of the average distribution of materials over the length of the structure. Ngai [102] stated that three different elements, namely weave design, fibre linear density, and the fibre bundle spacing must be carefully chosen to ensure optimum fabric and composite properties to produce the woven fabric reinforcement that fulfills the desired requirement.

The effect of yarn linear densities (276, 41.34, and 759 tex) of woven kenaf/unsaturated polyester composites on the mechanical properties of PE composites was studied by Saiman et al. [103]. They found that the mechanical properties of the natural composite were increased by the high linear density of the yarn. The greater linear yarn density indicated a greater fibre load within the composite in comparison to the composite’s mechanical properties (flexural and effect strengths). However, the increased linear density of the fabric would increase the composite’s thickness and weight.

Different linear yarn densities examined in another study by Madsen et al. [104] resulted in the different yarn structures, twisting angles, fibre orientations, the compactness of yarn, sorption characteristics, and mechanical characteristics of hemp yarn. They used two batches of hemp yarn, He47 (47 tex) and He53 (53 tex) and found that there were several findings with the characterisation of textile hemp yarn. These findings are important when the properties of composites with hemp yarn are predicted and interpreted. Due to the rise in fibre twisting angle, hemp yarn with greater linear density has a lower yarn diameter. The evidence of yarn cross-sectional area (which includes fibre and fibre spacing) was higher in yarn with low linear density. This defines the degree of compactness of the yarn, an important parameter in the estimation of the permeability of the yarn to matrix impregnation during composite manufacturing.

In terms of composite reinforcement, the twisted structure of fibre yarns is an important feature. This implies that the direction of the fibre in oriented plant yarn composites is governed by the angle of twisting of the fibre in the yarn. The twisting arrangement of the plant yarn depends on the method of spinning applied during yarn production. Better resistance to axial shear forces is the main benefit of the twisted yarn structure in these composites, though a loss of axial tensile properties has resulted from too high twisting angles. In this analysis, due to the loose structure of the yarn which provides less moisture permeability, the moisture sorption properties are measured higher in He47. The tensile properties of the hemp yarn are strongly affected by the conditioning humidity and the moisture content of the fibre. Low linear density yarn is loose in the structure and has led to increased sorption of moisture content. In the cell wall, the original intermolecular hydrogen bonds are interrupted, leading to increased mobility of the polymer chains. In addition, it is understood that the outer layer of the cell wall (the middle lamella) that bonds single fibres in bundles together is weakened by moisture. The decrease in apparent stiffness, the increase in ultimate stress, and the increase in strain at the final stress of yarn might be explained by these plasticization effects of moisture. They also concluded that tensile characteristics and moisture sorption capability are more important than other properties between the two batches of hemp yarn.

The twist is inserted in the fine strand of the fibre in the production of staple fibre yarns to hold the fibre together and to impart the desired characteristics to the twisted yarn. The fine fibre strand would be extremely weak without twisting. A transformation in the twist level also changes several yarn properties, including strength and softness properties. Two kinds of twists exist; (1) real twist and (2) false twist. In addition, one end of the yarn should be turned relative to the other end to insert a real twist in the length of the yarn. Spun yarn generally has a real twist that holds the fibre in the yarn together. When the false twist is placed in the length of the yarn, both ends, usually rollers, are clamped and the twist is placed between the clamping points with a false twister. If the yarn is not crossing its axis, the twist is above and beneath the false twister in opposite directions. If a false twister has been removed, the opposite twists cancel each other, leaving no real twist over the yarn’s length. If the yarn goes through the axis, it will have no net twist on the segment of the yarn moving away from a false twister.

**Figure 4:**
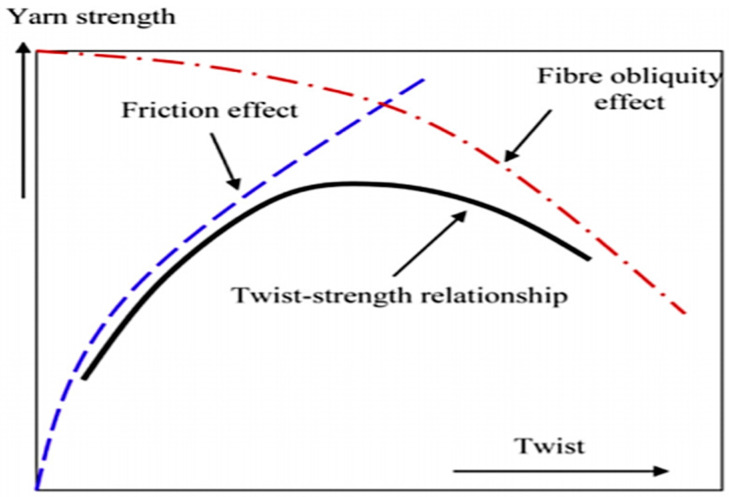
Effect of twist on the strength of yarn. Source: Figure reproduced with copyright permission from Miao et al. [105].

Twisting can be carried out in the following two directions: S and Z-direction. A single yarn has a “S” twist if the fibre inclined to the axis of the yarn conforms to the central part of the letter S in the direction of the slope as it is placed in a vertical position. A single yarn has a “Z” twist if the fibre inclined to the axis of the yarn conforms to the central portion of the letter Z in the direction of the slope when it is placed in a vertical position. There are so many factors that influence the properties of the yarn, including twist level and twist angle, especially the strength of the yarn. The twist degree is generally reflected in the number of turns per metre (tpm). More twists give any applied tension a greater radial component and improve the fibre’s resistance to slip. The quality of yarn improves as a consequence. In Figure 4, this phenomenon is represented by the “coherence curve”.

On the other hand, the twist will place the individual filaments under torsional stress if a bundle of parallel filaments is twisted. This tension weakens the filaments, and as the twist level increases, the strength of the filament will decrease. This is expressed by the curve of obliquity. For staple fibre yarns, as seen by the heavy line, these two curves combine to provide the actual twist-strength curve for a staple fibre yarn. The figure also shows that raising the twist level will increase the strength of the yarn to a maximum level for staple fibre yarn, above which a further increase in twist will decrease the strength of the yarn.

**Figure 5:**
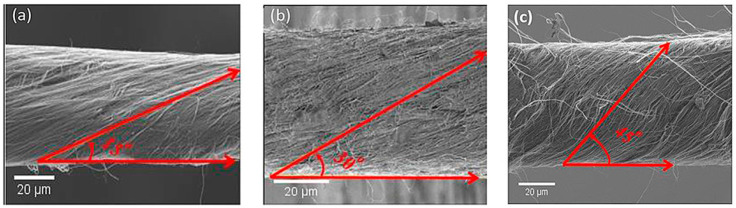
Yarn with different twist angles: (**a**) 15°; (**b**) 30°; and (**c**) 45°. Source: Figure reproduced with copyright permission from Anike et al. [106].

The twist angle is the angle of the fibre to the yarn axis, and this angle ranges from zero to middle to maximum on the yarn surface in the yarn. The fibre on the surface of the yarn is the most crucial, as the others are bounded into the yarn. A very important parameter is the surface twist angle created by the surface fibre relative to the yarn axis. It defines the essential characteristics of yarn, such as yarn softness, yarn bulk, etc., which in turn regulate many of the essential properties of the fabric. As shown in Figure 5a–c, the yarns have different twist angles of 15,30, and 45 degrees, respectively. This will suggest that the surface twist angles of each yarn are distinct. Regardless of the difference in yarn thickness, they will better represent the yarn characteristics.

Tensile strength is based on the maximum load a yarn or fabric specimen can endure when subjected to tensile loading, according to Realff et al. [107]. Taylor [108] claimed that the most significant property of a woven fabric is tensile strength that made woven fabrics differ from a non-woven or knitted fabric. Thus, demand for minimum fabric strength is applied to the normal structural details of fabric to ensure the consistency of fabric, yarn, and also fibre.

One of the key factors that might affect yarn strength (other than linear density) is the amount of twist. Low amount of twisted yarn has low tensile strength due to non-existent friction between the fibres. Yarn with a high amount of twists has high tensile strength, but a very high amount of twisting would also have a low strength due to the obliquity of the fibre. It is also crucial to know the optimal amount of twist to ensure that the entire strength benefits from inter-fibre friction and fibre alignment are maximum. Besides, since composites depend on the transfer of load between the fibre and the matrix via interfacial bonding, the role of twist in yarns becomes crucial because the strength of the yarn depends on the amount of twist.

The effect of the amount of twist on the flax yarn composite was investigated by Goutianos et al. [109]. Their study revealed that the permeability of the yarn decreased by increasing the level of twist and the tensile strength was diminished due to the increase in fibre obliquity, thus disturbing the efficiency of orientation. In addition, a high twist not only resulted in poor tensile strength characteristics but also inadequate wettability and poor fibre-matrix adhesion. Shah et al. [110] found that the strength and stiffness of the yarn improved with the rise of the twist to a pronounced peak, and the strength decreased as the twist expanded further. There are two factors that influence the strength of a yarn: (i) inter-fibre friction and cohesion, and (ii) fibre strength contribution in the loading direction. While the low twist yarn is usually more in line with the loading direction, fibre slippage occurred due to lack of inter-fibre friction.

There are many fibre properties that are important to be taken into account as they influence the final product to a large extent. Fibre length is one of the most important characteristics that determine the consistency of yarn, and its ability to twist. The length of most natural fibres ranges from ¼” to about 3”. In the case of silk fibre, it has a continuous filament shape that can be up to 2 km length. All natural fibres are valued from their lengths apart from silk, the greater their value, the longer the length. The natural fibre is a staple fibre that was cut into short fibres and used in the production of yarn. Thus, since it has a longer staple length, fibre with a longer length is ideal for yarn development.

Staple fibre length plays an important part in the conversion to yarn. In general, due to the long fibre that will have to overlap at less frequent points than short fibre, longer individual fibres are simpler to convert into yarn. Therefore, it is possible to easily spin longer fibres into a yarn. Apart from that, the conversion of long fibre into yarn requires less twist than short fibre. The length of the individual fibres in the fibrous system influences their overlap, affecting the typical frictional surface, and thus the consistency that can be obtained by, for example, twisting the yarn. Long and high friction fibre help lower yarn twist, which is typically desirable, which also allows finer yarns to be produced. Variation in the length of fibre and delivery can also induce variation. When spinning shorter fibre, the yielded yarn would have poorer strength due to the presence of short fibre. The involvement of short fibre influences, to a very large degree, the strength of the yarn. In general, fibre length is an important feature, since it affects the strength of the yarn, evenness, hairiness, and quantity of waste. Fibres below 4–5 mm will be lost when treated as waste, fibre sup to 12–15 mm will not contribute much to the strength but only to the fullness of the yarn. Only fibres over 15 mm in length produce yarns with desirable features.

Natural fibres have typical shapes and a significant variation in their cross-sectional form [111]. Cotton, for example, has a bean shape that differs due to external factors, such as maturity. The cross-sectional form of man-made fibre is influenced by the extrusion method, the spinneret used and post-processing to a certain degree by the spinning design. Symmetrical cross-sectional shaped fibres also exhibit symmetrical mechanical bending behaviour. Bending deviates from those in circular cross-sections with asymmetric cross-section forms, influencing bending deformation and torsion. The contrast between hollow and solid fibre revealed that circular and shaped fibres appear to lead to stronger fabrics when used in hollow round fibres [112]. Cross-sectional types also affect the fibre surface area and can represent inter-fibre friction.

The density or volumetric mass density is its mass by unit volume (g/cm^3^). The total mass is taken into account in measuring fibre density because of external variable and conditions, such as moisture or impurity in natural materials. The impact of measurement conditions is usually minimised using standard conditions and conditioning. The weight of all the fibres together along with yarn or fabric is determined by the density of the fibre of which it is produced.

Crimp could be seen as the hairiness of yarn and it is the key characteristic that regulates the bulkiness and basic volume of yarns and fabrics [113]. Fibre crimps, whether in two or three dimensions, can be described as waves, bends, twists, or curls along the fibre length. It is presented as crimps per unit length and might be natural, as it is artificially imposed often with natural fibre. The stress-strain curve of the fibre usually consists of three separate mechanisms: removal of slack, removal of crimp, and stretching of the fibre. Crimp removal is defined as time due to empirical data occurring below 1 cN/tex, but later studies have shown that the definition of the region is more complicated. More accurate results for the calculation of the crimp can be obtained by identifying the slack and tensile regions and by deducting the crimp area from the data [114].

The volumetric properties or the volumetric bulk are influenced by crimp, affecting haptic properties. Before the fibre is straightened and tensile properties are involved, it can also serve as a buffer under stress. Crimp improves the cohesion of fibres, which is beneficial, for example with slick fibres, and even the tensile strength and compression behaviour of a fibre assembly [115].

The fineness of the fibre is also an important parameter regulating the twisting capacity of the fibre to be transformed into yarn. A finer individual fibre generates afiner yarn that has more fibre per unit of cross-section compared to the coarse fibre yarn. This greatly increases the cohesion between the fibre inside the yarn. In addition, the fine fibres appear to be more flexible and foldable than the coarse ones, allowing it to be more easily spun into the yarn. The calculation of the fineness of the fibre, which is how dense or thin is defined in terms of the number of fibres. The direct method of determining the fineness of the fibre count is to describe the thickness or diameter of the fibre in microns, where 1 micron = 0.001 mm. The fibre count is defined in terms of mass per unit length, i.e., linear density. The linear density is defined in terms of a milligram per kilometre (millitex unit), gram per kilometre (Tex unit), and gram per 9000 m (Denier unit).

**Figure 6:**
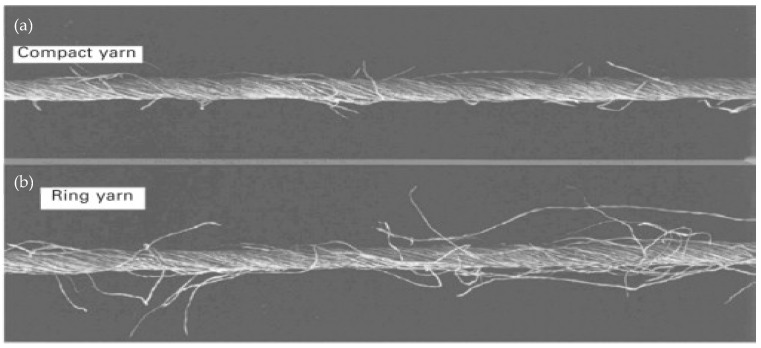
Yarn structure of (**a**) ring yarn and (**b**) compact yarn. Source: Figure republished from El-Sayed and Sanad [120] with permission from Elsevier.

Various terms are also used to define yarn compaction, such as packaging fraction and packing density. The packaging density could be defined as the fibre volume ratio with the yarn volume, the yarn density ratio to the fibre density, and a total cross-sectional fibre area ratio to the cross-sectional area of the yarn [116]. The packing fraction is an indicator of the area of the air covered by the fibre. Numerous researchers have shown that compact yarns have improved properties in several ways, as the fibres in compact yarns are almost fully integrated into the yarn body [117,118]. On the other hand, Basal [119] suggested that for a compact yarn, migration exists at higher levels and leads to a greater structure and consistency of the yarn. Lower yarn hairiness, higher strength, and elongation values are the primary benefits of compact yarns. Other benefits of compact yarn are: (i) lower amount of dust removed due to the more compact structure of the yarn, (ii) greater efficiency associated with higher tensile strength, (ii) lesser hairiness and higher tensile strength significantly reduces yarn breakage by 30% during warping, (iv) lower yarn breakage (by 30–60%) during the spinning process, (v) greater break tenacity and elongation by 8–15%, and (vi) lower irregularity of mass. Compact yarn or yarn with a greater packing density usually has a lower yarn diameter and a higher number of contacts between fibres as shown in Figure 6.

A high packing fraction produces stronger and less hairy yarn, resulting in high fibre-fibre interaction, lowered wettability, and reduced intra-yarn adhesion in composites. As a result, the packaging fraction serves a crucial role in the final composite features. Pan [121] and Cox [122] have proposed that fibre-to-fibre spacing becomes so small at high packing fractions that the stress transfer between fibre and matrix gradually becomes ineffective, cause early failure due to higher shear stresses on all planes parallel to the fibre axes. Rasyid et al. [123] argued that fabrics with lower inter-yarn fabric porosity contribute to greater penetration resistance because higher forces are needed to penetrate tight-structured fabrics. This will affect the penetration of the matrix during the composite manufacturing process.

### 3.2. Fabric Properties

**Figure 7:**
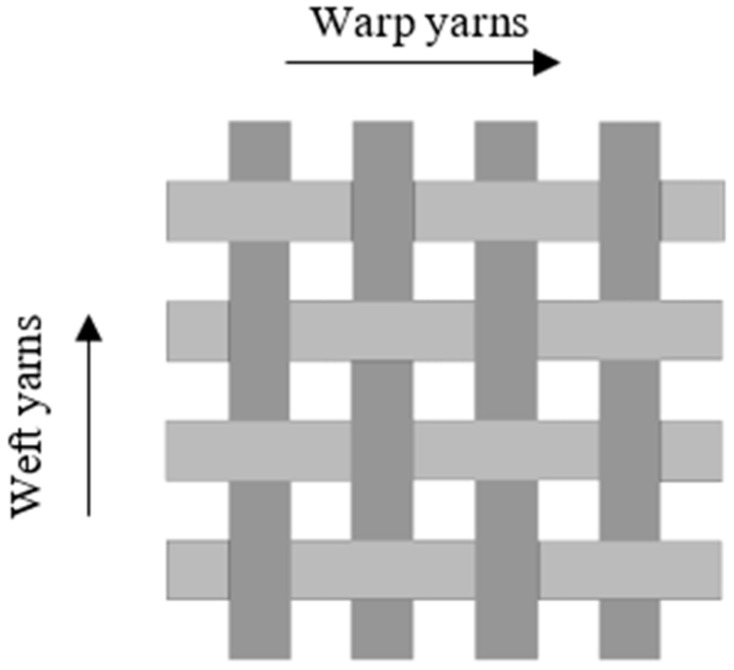
The warp and weft yarns in the fabric structure.

In general, woven fabrics comprise of two sets of interlaced yarns that lie at right angles to each other. The threads running along the length of the fabric are referred to as warp ends, while the threads running from selvedge to selvedge, which is from one side of the fabric to the other side, are weft picks, as seen in Figure 7. The inherent benefits of woven fabric composites increase their utilisation in advanced structural applications. In engineering, the key benefit of using woven fabric laminated materials is that they have more balanced properties at a temperature of 0 °C and 90° directions than unidirectional laminates. There are numerous other benefits of laminated materials in textiles, including ease of handling for automation, compliance with complicated forms, thicker fibre shapes, and improved toughness of fractures [124]. Kelly [125] has noted that very rigid tissue fibre has a long-term engineering perspective.

Woven fabrics form, in particular, through the interlacing of yarns into a fabric layer offers good dimensional stability and high packaging density benefits. The vertical direction of the yarn is called the warp, while the horizontal direction of the yarn is called the weft. The use of a woven technique might add structural strength to the material because it raises both the strength and the energy absorption capacities. Woven fabrics are used in a broad spectrum of protection and consumer goods as a reinforcement process of composites due to their durability, formability, and high specific strength, as interlocking improves strength to be stronger than fibre matrix adhesion [126]. Yarn slip is an essential deformation or fault mode that causes drastic changes in the energy absorption and the yarn density of fabric to become relatively sliding of the yarns composing the weave.

The fabric is created by means of textile methods of braiding, knitting, sewing, or weaving. Of these methods, weaving is one of the most widely used in the manufacture of composite fabrics, due to high productivity combined with flexibility in the production of a wide variety of fabric structures. Woven fabrics are desirable as reinforcements as they have outstanding integrity and conformability. Woven fabrics are produced in a standard pattern or woven structure through the interlacing of warp fibre and weft fibre. Warp yarns run along the length of the fabric, while weft yarns run perpendicular to warp yarn.

Because of the morphological properties of the fibre, fabrics from different fibre sources have developed different properties of the fabric. For example, research performed by Morris et al. [32] found that treated fabrics made of bamboo, lyocel, silk and cotton fibres reinforced with epoxy and mixed with ultraviolet (UV) photocuring soya resin resulted in various behaviour of the final composite for medical imaging application. They discovered that the highest flexural modulus is provided by cotton reinforced epoxy composite, but bamboo reinforced epoxy composite provides minimum X-ray attenuation without major flexural modulus compromise.

**Figure 8:**
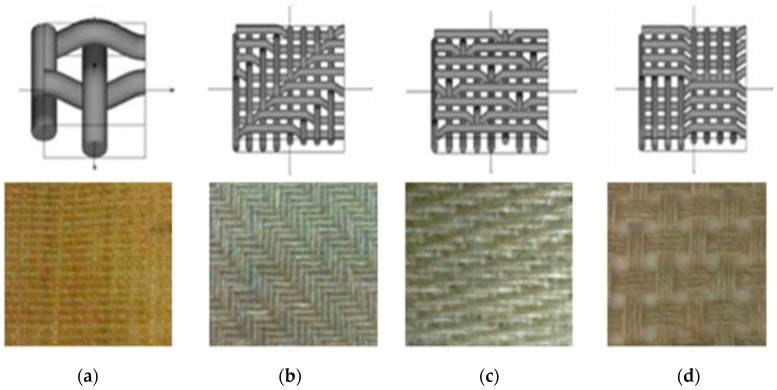
Woven fabric from kenaf yarn with 3D images from WiseTex software (**a**) Plain, (**b**) Twill, (**c**) Satin, and (**d**) Basket. Source: Figure reproduced with copyright permission from Othman et al. [127]. This article is an open-access article distributed under the terms and conditions of the Creative Commons Attribution (CC BY) license (http://creativecommons.org/licenses/by/4.0/).

A few woven structures such as plain, twill, satin, basket, leno, and mock leno are being used in the fabric construction. In textile composite applications, however, there are three different types of weaves known as basic weaves, i.e., plain, twill, and satin (Figure 8). The most common woven structure used in the development of composites consists of the plain weave. The simple, smallest repeat sized possible, easier to form, and easier to recognise, plain weave is the basic reinforced structure of a textile composite.

Each warp fibre alternately passes under and over each weft fibre in the plain weave structure. The fabrics are symmetrical, stable and of reasonable porosity. It is however the hardest weave to drape, and the high fibre crimp levels have relatively low mechanical characteristics in comparison to other weave styles. This weave style is excessively crimped with large fibre (high Tex) and therefore, does not tend to be used in very heavy fabrics. In the twill weave structure, one or more warp fibres alternately weave over and under two or more weft fibres on a daily basis. This results in the visual effect of a straight or broken diagonal “rib” on the fabric. Superior wet out and drape were shown in a twill weave over a plain weave with only a slight decrease in stability. Compared to plain weave, the twill weave structure is more stable and has less crimping, and the fabric has a finer surface with slightly higher mechanical properties. The twill woven has a looser structure and is distinguished by floated yarns forming a diagonal line. It also has greater drapability and is more stable compared to satin weave.

Satin weaves are essentially twill weaves adjusted to create less warp and weft intersections. The number of “harnesses” used in the designation (usually 4, 5 and 8) is the total number of fibres crossed and passed down before the fibre repeats the pattern. A “crow’s foot” weave is a form of satin weave with a different stagger in the pattern of repetition. Satin weaves are very flat, well wet out, and have a high level of drape. Good mechanical properties are given by the low crimp. Satin weaves enable the fibre to be woven close to each other and can create fabrics with a close “tight” weave. Although the low stability and asymmetrical of the structure must be considered, asymmetry allows one face of the fabric to have fibre running mainly in the warp direction, while another face has fibre running mainly in the weft direction. Satin weaves have a smooth surface and many floated warp yarns producing good flexible drapability, which is usually used to form curved surfaces. Table 3 shows a summary of the comparison between the properties of the fabric for each woven structure.

Some characteristics of woven fabrics are appropriate for applications requiring high strength, modulus and impact. The strength of tissue is one of the most essential features to make it preferable in ultimate applications. The strength of the fabric is usually caused by converting fibre resistance to yarn strength and strength in the fabric. Nevertheless, various factors such as fibre type, yarn sizing, fabric count, or weave structure can influence the strength of the fabric. Numerous researchers have shown that the strength of the fabric depends not just on the yarn’s strength, but also the yarn and fabric structures [103,107,128].

Saiman et al. [129] mentioned that, rather than a weave structure, yarn crimps have a major influence on the textile strength, in line with Ferdous et al. [130] and Maqsood et al. [131]. The waviness of warp yarn and weft yarn that interlace in a fabric together is yarn crimp. Causa and Netravali [132] noted that the frictional resistance between the fibres takes place first, followed by the interchanged crimp then the extension of the yarn where the load is applied towards to the yarns, when the load is applied to a woven fabric. Based on Peerzada et al. [133], if a load is applied to a woven fabric and the yarns are uncrimped, the maximum load at full strength would be met with stress. If the yarns are crimped, however, the initial load will be consumed in the straightening of bent tows and the load will then be taken up, resulting in low strength properties.

The findings from Saiman et al. [129] were however, in contrast between different kenaf fabric weaving structures as a structural material for the manufacturing of natural composite-based materials by examining the relationship between the yarn crimp and the stability of the fabric tensile strength. In the analysis, it was found that the plain weave had the highest crimp percentage accompanied by twill and satin weaves. Plain weave is very crimped due to the higher interlacing of yarn comparison to other weaves. Less crimp offers a higher strength of the fabric due to its free inter yarn and fibre that contribute to the force. In this study, it was found that the satin fabric had the highest strength followed by twill and plainness. The largest proportion of crimp in plain fabric had the lowest strength. The fabrics performances of kenaf and cotton yarn with plains, twill, and satin weave structures were evaluated by Ramaswamy and Easter [134]. They found out that the strength of the fabric was influenced by the structure of the woven, which the satin weave was stronger than the twill weave.

According to Ferdous et al. [130], twill weave provides excellent strength in comparison to satin and plain weaves. This is because when the woven fabric is stretched, the crimp in that direction starts to decline. Fabric was comparatively easy to build when the crimp was weakened. Then, the yarn started bearing the load that trimmed down the extension tempo of the fabric. The strength of the fabric was lower than the strength of the twisted yarn due to the crimp; the strength of the yarn was lower than the strength of the fibre because of the twist in the yarn. Although plain weave had a higher interlacement, the maximum crimp was found in plain weave. Thus, there was only a small amount of fibre slippage in the yarn. This was the explanation of low strength shown by plain weaves. Banerjee et al. [135] also performed a study on the impact of woven structure (plain, twill, satin) on cotton yarn woven fabrics and noticed that the strength of the fabric was significantly affected by the values of crimps. Higher crimp values, but higher elongation, led to a lower strength.

Modulus is greatly influenced by the composition of the weave. Ramaswamy and Easter [134] showed that the elongation at break of woven kenaf/cotton fabric varied from 35%, 21%, and 19% for plain, satin, and twill woven fabrics, respectively. The results of Banerjee et al. [135] also exhibited that the structure for the plain weave had the highest modulus than in satin and twill weave. They proposed that the modulus and elongation of woven fabrics were affected by the degree of interlacing between yarn and the state of relaxation of the fabrics. Due to the frictional force of the yarn to the yarn contact points, the relaxed fabric had steeper load-elongation curves.

The load-extension curve in the initial decrimping region has an inflexion point, probably, the initial high modulus of the fabric was due to the frictional resistance of the yarn bending. After overcoming the frictional restraint, a relatively low modulus was generated that was mainly governed by the strength required to unbending the yarn in the direction the force used and at the same time, the twist of the yarn at the right angles in the direction of force application needs to be increased.

The structure of the fabric and the mechanism of energy absorption of the fabric are important woven fabric parameters for the impact properties. In addition to the high strength yarn used for impact applications, the fabric weave also provides penetration resistance. The number of yarns or fabrics counts in the fabric refers to the cover factor, whereas, it is proportional to the energy absorbed. Fabric count is the number of yarns in 1 cm (yarn per cm). Fabric count is essential as it is responsible for the tightness of the woven structure.

The cover factor (CF) is defined as the ratio between the area covered by the yarn and the overall fabric area [136]. CF shows the extent to which one set of yarn covers the area of a fabric [137]. CF might be regarded as the fraction of the total fabric area covered by the component yarns for fabrics constructed from yarns. Milasius [138] and Milasius et al. [139] indicated that CF is a basic construction parameter of woven cloth related to its performance in end-use. The efficiency of weaving, fabric quality, and air permeability are the characteristics closely that are correlated to CF. Plain weave has the greatest number of intersections per unit area. Some weaves have fewer intersections than the plain weave. The probable weaveability of all fabrics woven with the same weave and related yarns can be predicted from their CF. Two main components of CF are the fractional cover and the absolute cover. Higher fractional coverage suggests a low gap between warp and weft yarn. As a result, fabric with high stability and balance weaving structure is made.

**Figure 9:**
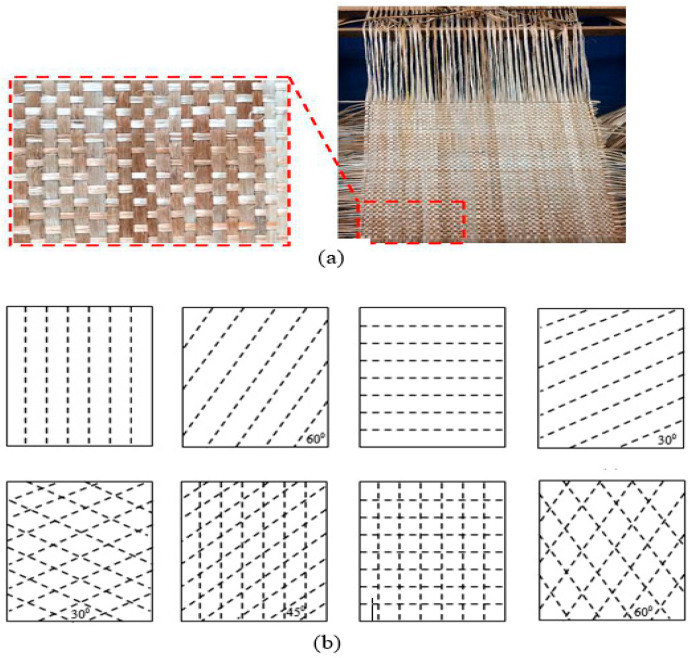
(**a**) Woven fabric from kenaf yarn with plain weave pattern (**b**) types of stitch patterns used in this study. Source: Figure reproduced with copyright permission from Yaakob et al. [92]. This article is an open-access article distributed under the terms and conditions of the Creative Commons Attribution (CC BY) license (http://creativecommons.org/licenses/by/4.0/).

There are a number of fabric modifications to improve the fabric properties of NFPCs. One typical aim is to improve the properties of the fabric by changing the woven structure. Alavudeen et al. [78] examined the mechanical properties of banana and kenaf fibres composites with weaving pattern effects and random orientation. The study found that composites with a plain weave structure provided better mechanical strength compared to a random composite orientation, suggesting that the woven structure affected the mechanical properties of the composite. Yaakob et al. [92] compared the inclusion of stitches on the woven kenaf fabric in other studies in order to increase the strength of the through-thickness, the resilience of the interlaminar fracture, and the tolerance of impact damage between the polymer matrix and reinforcement. On the fabric of the kenaf woven, as shown in Figure 9, eight types of stitching patterns focusing on stitch patterns and angles were used. They concluded that the addition of stitch increased the composite specific power, with stitching patterns and stitching angle having a significant impact compared to the unstitched on the output of woven stitch kenaf composite.

**Figure 10:**
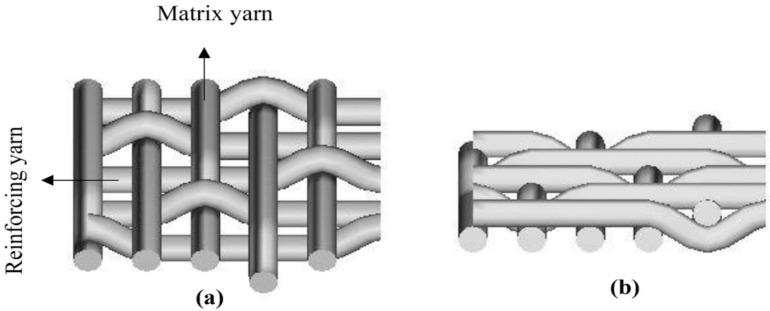
Schematic representation of comingled woven fabric (**a**) top view (**b**) side view. Source: Figure reproduced with copyright permission from Awais et al. [95] with permission from Elsevier.

**Figure 11:**
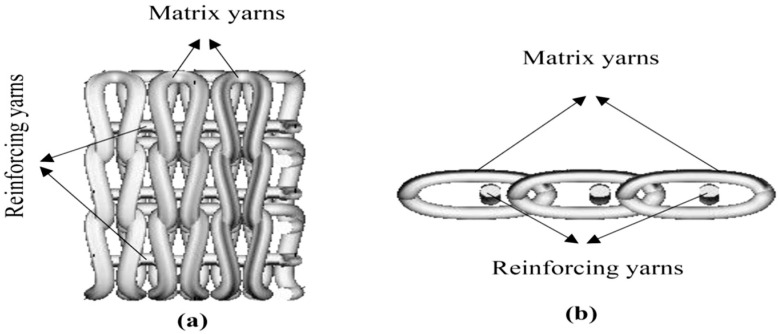
Schematic representation of comingled knitted fabric (**a**) top view (**b**) side view. Source: Figure reproduced with copyright permission from Awais et al. [95] with permission from Elsevier.

Awais et al. [95] also examined another approach to the use of fabric alteration. In this study, three different commingled fabrics were made from jute, hemp, and flax yarns in the warp direction and polypropylene yarn in the weft direction by weaving (Figure 10) and knitting (Figure 11). Essentially, the fabric made of yarns usually contains both reinforcement and matrix in the form of fibre, used in the manufacturing of reinforced fibre composites. The results demonstrated that the mechanical characteristics of the composite, specifically the tensile, flexural, and impact characteristics of knitted commingled composites, were greater than those of woven commingled composites. These properties for flax composites, in contrast, were superior to those for both knitted and woven composites for hemp and jute composites. Derbali et al. [140] also produced laminate composites of flax and polypropylene fabrics with different weave designs, with the main aim of minimising the process steps and shortening the production time required. In this study, optimum process parameters, such as pressure level, temperature, holding time, and cooling rate, were acquired for the production of composites with small voids and good strength.

### 3.3. Composites and Manufacturing Parameters

Numerous studies have reported results on several factors influencing natural fibre woven composite physical and mechanical properties. The factors included: (i) composites parameters such as fibre sources, reinforcement types, reinforcement quantities, laminate sequence, fibre loading percentage amount, fibre orientation, laminate layer numbers, treatment, and (ii) production parameters such as processing technique, temperature rate, pressure level, time to hold, etc.

Natural fibres are well known to be hydrophilic in nature, produced in a fibre structure by the hydroxyl groups. In contrast, the matrices are generally hydrophobic and therefore, due to the non-uniform fibre dispersion in the matrix and poor fibre-matrix interface, the combination of these materials are not compatible. Fibre treatment is, therefore, one of the solutions for enhanced fibre surface area, chemical bonding, and interface adhesion between matric-natural fibre [52,53,54,141,142,143,144,145,146,147,148,149]. The effect of treated woven aloe vera and flax soaked in 5% potassium permanganate (KMnO_4_) and 10% sodium hydroxide for 1 h and filler with sulphate barium sulphate (BaSO_4_) was studied by Arulmurugan et al. [150]. Three different fibre sequences for composite specimen were prepared using the lay-up technique and the mechanical characterisation of woven fibre-reinforced epoxy polymer composites was evaluated. The results showed that the tensile strength of the composite consisting of four layers of woven flax increased with the addition of BaSO_4_ due to fibre treatment, a more appropriate mixture of matrix and filler, as well as improved interlacement in the flax fabric that created interlocking in the fabric structures between the fibres. This form of composite also displayed a higher strength of hardness due to the denser mat structure of the flax fabric. However, composites with only aloe vera fabric with no addition of BaSO_4_ resulted in stronger impact strength. This form of composite absorbs the energy of impact that efficiently attributed to the versatility of the aloe vera fibre fabric.

**Figure 12:**
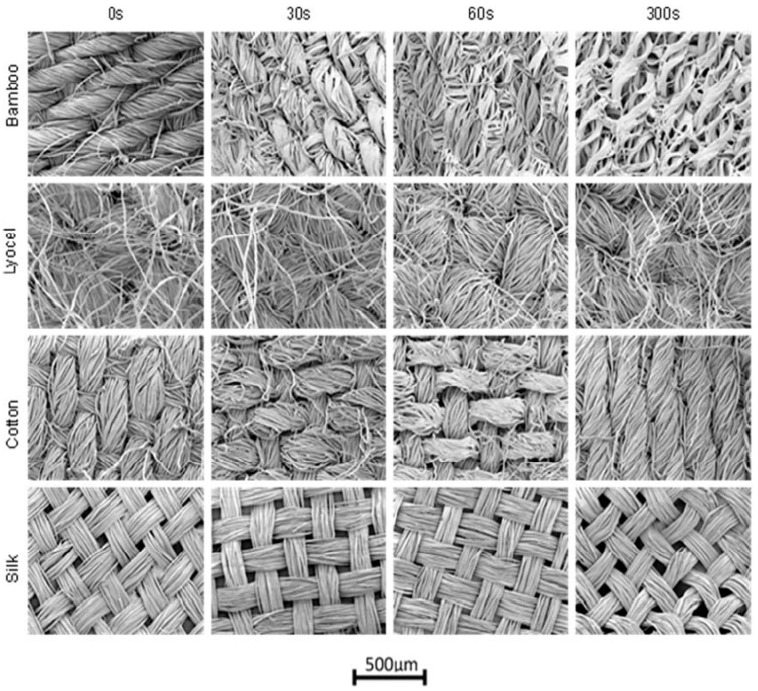
Micrograph images of woven silk, cotton, lyocel, and bamboo after immersion in sodium hydroxide solution for several times interval. Source: Figure reproduced with copyright permission from Morris et al. [32]. This article is an open-access article distributed under the terms and conditions of the Creative Commons Attribution (CC BY) license (http://creativecommons.org/licenses/by/4.0/).

In another study, Morris et al. [32] studied the effect of using the solution of sodium hydroxide to change the surface structure of woven silk, cotton, lyocel, and bamboo as a result of soaking time on the mechanical properties of the medicinal application of the epoxy-based composite. Figure 12 shows the topography of the surface and images in the morphology of woven fibre after treating in the NaOH solution for 30 s, 2 min, and 5 min of immersion time to demonstrate matrix evolution over time. The resulting properties of composite materials were found to have minimal influence on the structural change of the composites. The treatment, however, did not prove that the final composite materials had improved the strength or image of the composites.

Hybrid aloe vera, sisal, and flax reinforced epoxy composites developed by Balasubramanian et al. [151] were therefore distinguished through tensile, flexural, impact, and hardness tests. The principal parameters were the sequences of aloe vera and flax mat. There were two types of composite with different fibre mat sequences; S1-with arrangement of aloe vera mat/flax mat/sisal fibre/flax mat/aloe vera mat, and S2 with the arrangement of flax mat/aloe vera mat/sisal fibre/aloe vera mat/flax mat. The findings indicated that hybrid composites formed with flax fibre mat positioned on the outer layers (S2) had higher tensile strength, flexural, impact, and hardness than hybrid composites from aloe vera fibre mat on the outer layers (S1). This could be attributed to the properties of flax fibre. They also concluded that the positioning of the fibre mats was one of the roles in determining the composite properties. Awais et al. [95] investigated natural fibre composites including jute, hemp, and flax, reinforced via the compression moulding technique with polypropylene matrix. Three distinct fabric architectures were developed using jute, hemp, and flax yarns, such as woven, woven commingled, and knitted commingled fabric. They evaluated the shear and impact strength of hybrid composites’ fabric architecture. The results indicated that shear strengths were enhanced for all types of natural fibres in the knitted commingled composites compared to woven composites and woven commingled composites. However, interesting findings were obtained, where woven composites exhibited improved performance in terms of impact resistance compared to woven commingled composites and knitted commingled composites, which contributed to the waves of the fabric woven structure and the interlacing pattern which improved the damage resistance properties of woven laminates.

The effect of hybridisation of woven flax and carbon fibre formed by vacuum-assisted resin infusion on the tensile properties was explored by Kureemun et al. [88]. There were five types of hybrid flax-carbon stacking sequences reinforced with epoxy. The results of their findings showed that the tensile properties of the multi-carbon hybrid composite with a flax ply had improved tensile steadiness by nesting the rigid woven carbon plies and better architectural crimps between the woven fabrics of flax and carbon. Torres et al. [152] produced jute and flax woven fabric composites in a variety of material and geometric configurations, with the aim of determining statistical distributions of mechanical properties. The composites were manufactured by vacuum-assisted resin transfer moulding (VARTM) with epoxy as a matrix. Several weave designs were selected namely plain, twill, and satin, and geometrical configuration for long fibre were 0°, 90°, and 0°/90° directions. A statistical analysis of the elastic modulus, strength, and failure strain of the composites for woven fabrics showed that the woven composites of both fabric types were stronger than the random arrangement due to the strength of the reinforcement fibre in the direction of loading. However, as compared to woven flax composite due to fabric crimps in the fabric structure, the strength of long flax with 0°/90° directions (non-woven) was found higher.

**Figure 13:**
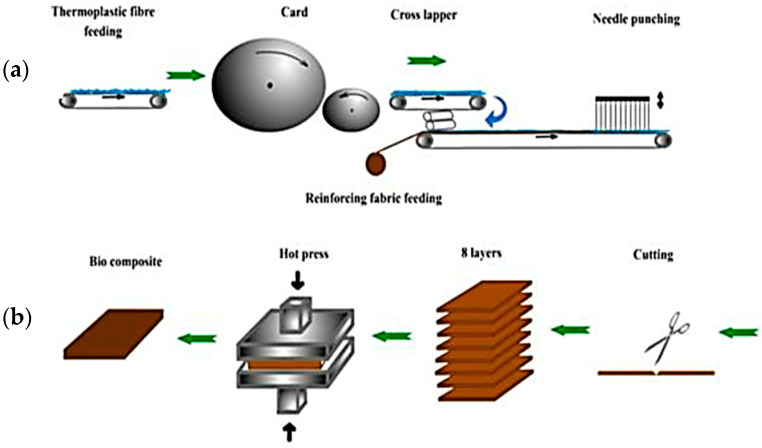
Graphic process of (**a**) hybrid woven fabric, and (**b**) composite laminates using hot-press technique. Source: Figure reproduced with copyright permission from Kandola et al. [87] with permission from Elsevier.

Kandola et al. [87] produced natural fibre woven composites fabricated from jute and sisal yarns. Hybrid woven fabrics were created using a needle-punching process with PP and PLA fibre webs with a woven fabric/thermoplastic fibre ratio of 40:60 (*w/w*) (Figure 13a). Woven fabrics were used in the fabrication of the composite laminates via hot-press technique (Figure 13b). The tensile, flexural, and impact measures were used to determine mechanical properties, while fire output was analysed by restricting the oxygen index, flame spread, and calorimetry of the cone. In both cases of jute and sisal weaved composites compared to the PP composites, improved tensile and flexural properties were observed in PLA composites. Sisal composites have higher tensile and flexural modulus than jute composites concerning the types of woven natural fibre used. Nevertheless, the impact properties of the PP woven composites had improved. Furthermore, PLA woven composites were found to be less flammable and less smoke-producing compared to PP woven composites.

Yallew et al. [153] analysed the effect of manufacturing parameters on the tensile strength of woven hemp, jute, and sisal reinforced polypropylene (PP) composites, namely the heating temperature and pressure. Using the compression moulding method with three separate heating temperatures (165, 175, and 185 °C) and compressive pressures (0.5, 1.0, and 1.5 MPa), composite specimens were prepared at a constant rate of curing time of 4 min. They found that composites manufactured using woven sisal at a temperature of 175 °C and a pressure of 1 MPa had the highest tensile strength characteristics compared to other conditions.

Sivakumar et al. [85] manufactured woven kenaf fabric (K) and glass fibre (G) composites using different fibre configurations, i.e., KGK and GKG, and matrix types, i.e., PP, epoxy. In this research, PP-based hybrid composites were produced using the hot press moulding compression process, while epoxy-based hybrid composites were manufactured using the vacuum-assisted infusion approach. Hybrid biocomposite tensile and fatigue strengths, as well as micrograph images of failure specimens, were evaluated. The strength and tensile of fatigue were improved with the increasing amount of glass fibre in the composite layer were found in both matrixes. Adding two layers of G enhanced fatigue in the composite of PP, but reduced tiredness at low load levels. The fatigue strength appeared to be significantly improved with epoxy as a reinforcement agent compared to PP for the same fatigue lifecycle. For the matrix used, composite with fibre configuration of GKG reinforced with PP resulted in highest specific fatigue strength than composite reinforced with epoxy, while for the fibre configuration of KGK, the highest specific fatigue strength at a low load level was also found in the PP-based composite.

## 4. Properties of Woven Natural Fibre Polymer Composites

### 4.1. Woven Polymer Polypropylene (PP) Composites

Several studies have been conducted on the characteristics of different woven natural fibre reinforced polymer composites. Yallew et al. [154] investigated the suitability of woven polypropylene reinforced jute (PP) composites for tribological components, such as surface flooring, automotive brake pads, shoe soles and artificial human joints. The effect of the reinforced jute fabric on the resulting frictional composite behaviour was investigated. They found that strengthening the composite with woven jute lowered the friction value compared with the neat PP. They also concluded that strengthening woven jute fabric in the PP matrix increased the wear resistance characteristics of PP-based composites. Therefore, the jute/PP composite is ideal for many applications in tribological components.

PP biocomposites were incorporated in a current follow-up study by Yallew et al. [153] on the effect of process parameters on the mechanical properties of compression moulded woven natural fibre (sisal, jute, and hemp). The results showed that the highest tensile strength (41.48 MPa) was recorded at 175 °C of heating temperature and 1 MPa of compressive pressure moulding conditions. The lowest tensile strength (33.25 MPa) was achieved at a temperature of 185 °C and a pressure moulding of 1.5 MPa. Sisal fibre is more effective than hemp and jute fibres as a reinforcement of bio-composites with regard to strengths. The morphological study showed the influence of the moulding process parameters on the tensile behaviour of the fabricated composites.

The highest tensile strength (41.48 MPa) was recorded at a pressure of 1 MPa pressure moulding and a heating temperature of 175 °C. The lowest tensile strength (33.25 mPa) was produced at a pressure of 1.5 MP and a heating temperature of 185 °C. Sisal fibre was more powerful than hemp and jute fibre as the biocomposites’ strengthening agent. The morphological study showed the effect of moulding process parameters on the tensile behaviour of produced composites.

Akonda et al. [155] studied the application of semi-consolidated tape in unidirectional and biaxial woven flax reinforced polypropylene bio-composites. The outcome revealed that the lightweight tape was structurally stable and had a volume of 38% flax fibres. The composites based on flax/PP tape had 35–65% higher tensile modulus and 60–110% higher flexural modulus compared to thermoplastics based on flax/PP yarn. For the semi-consolidated tape unidirectional composite (TUDC), 125 MPa and 132 MPa were the highest tensile and flexural strength obtained. Thermal results showed that under the heating conditions of the tape process, the flax fibre-reinforced PP matrix did not deteriorate. Akonda et al. [155] concluded that semi-consolidated flax/PP tape was a significant step in the improvement of natural fibre enhancement effect in composite applications when compared with yarn-based composites.

A study on reinforced polypropylene composites was conducted by Awais et al. [156] on natural fibre (jute, hemp, and flax). These natural fibre thermoplastic reinforced polymer composites are gaining interest for ideal materials in critical weight applications. The commingled fabrics has evolved as alternative ways to resolve the viscosity constraint and reduce the cost of production by means of faster lay-up. A comparative performance assessment was carried out in this research work on natural fibre-reinforced (jute, hemp, and linse) laminates fabricated using woven, woven commingled, and knitted commingled fabric architectures, together with a polypropylene matrix by compression moulding method. The damage behaviour of the manufactured laminates were evaluated with regard to short-beam shear (SBS), the strength of drop weight impact, and compression after impact (CAI). The result showed that knitted commingled laminates had higher SBS strength (up to 29% and 20%) and compression following impact (up to 37.9% and 25.3%) compared to woven and woven commingled laminates. The woven laminates, however, provide a higher impact resistance due to fibre interlacing compared with the knitted and woven commingled laminates.

Currently, there are relatively limited extensive studies of the commingled techniques for continuous yarn. In a further study by Awais et al. [156] on the commingling techniques effects on mechanical properties on natural fibre strengthened cross-ply thermoplastic composites, the tensile, bending, and impact proprieties of knitted commingled laminates were higher than those of woven commingled laminates. Knitted and woven commingled fabrics were fabricated using novel commingling methods in this experiment, and then using these fabrics the composite manufacturing was carried out. During the manufacture of thermoplastic composites, the impregnation of resin into the reinforcement was seen as a significant concern. As a result, intermediate materials known as commingled fabrics were produced to assist in the manufacturing of continuous fibre reinforced thermoplastic composites by aligning polypropylene fibres with natural fibre reinforcements (jute, hemp, and flax) using weaving and knitting techniques. Cross-ply composite panels were produced using the hot press moulding process. The mechanical characteristics of knitted commingled composite specimens were superior to those of woven commingled composite specimens. The experimental results showed an increase of 14%, 7%, and 3% in tensile strength, 25%, 20%, and 13% in flexural strength and 37%, 54%, and 44% in impact strength of jute, hemp, and flax knitted commingled specimens, respectively.

The experiment on the matrix effect (thermoplastic polypropylene and thermoset epoxy) and glass/kenaf woven fibre configuration in the fatigue of composites performance was conducted by Sivakumar et al. [85]. Composites based on thermoplastic fibre-reinforced were fabricated using a hydraulic hot press machine through the compression of the hot press process. The [GKG] is referred to as the hybrid composite or the outer layers were substituted by a glass fibre fabric, while the hybrid composite, the middle kenaf layer was substituted by one glass fibre layer is referred as [KGK]. The effects of matrix, PP, and epoxy on their fatigue properties were investigated, not only for the different fibre configurations. The results of the work showed that the increase in tensile strength and fatigue was due to the considerable quantity of glass fibres present in the laminate. Increased fatigue sensitivity in the polypropylene-based laminate by introducing two layers of glass fibre reduced the fatigue strength of the laminate at a low load level. By contrast, a hybrid composite based on epoxy had lower fatigue sensitivity. Overall, these findings show that PP-based hybrid composites had increased fatigue resistance relative to epoxy-based hybrid composites. It can be noted that PP-based composites were less sensitive to fatigue loading, as evidenced by the enhancement of the thermoplastic matrix. In terms of specific fatigue strength when density was taken into account, [GKG] reinforced PP displayed the highest specific strength due to higher epoxy density compared to [GKG] reinforced Epoxy, whereas [KGK]/PP had a higher specific fatigue strength compared to [KGK]/E at a low load level.

**Figure 14:**
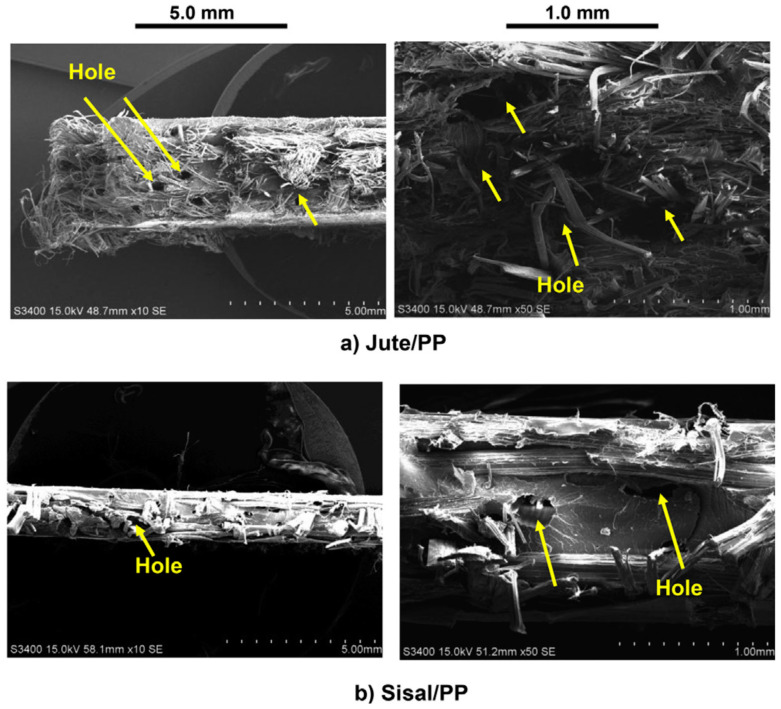
SEM images of the fractured surface of tensile tested specimens at two different magnifications of (**a**) jute/PP, and (**b**) sisal/PP. Source: Figure reproduced with copyright permission from Kandola et al. [87] with permission from Elsevier.

Kandola [87] recently studied the mechanical and fire properties of hybrid composites on the effect of natural fibre-reinforced thermoplastic composites from woven-non-woven textiles. The use of woven jutes and sisal materials to strengthen polypropylene (PP) and polylactic acid (PLA) matrices was developed for natural fibre-reinforced composites. It can be summarised that woven- and nonwoven technology can be used for the preparation of polymer composites. The natural fibre reinforcement also increased the mechanical properties and reduced the flammability properties of PP and PLA composites. Among PP and PLA composites, PLA showed better tensile and flexural properties, while its impact properties were lower compared to PP composites. In comparison with their respective PP composites, PLA composites were also less flammable and produced less smoke. The tensile strength values for sisal/PP and jute/PP composites with different reinforcements showed a similar pattern as for the respective PLA composites. The tensile modulus of sisal/PP (6.5 GPa) composites were observed to be higher than jute/PP (4.8 GPa). The holes formed by the fibre pullout from the jute/PP and sisal/PP fibres can be obviously seen in Figure 14a,b in the scanning electron microscopy images. This can be explained by the difference between each component’s chemical structures, where jute and sisal contained hydroxyl-group cellulosic fibres on the surface, and were therefore, poorly compatible with non-polar polymers, such as PP.

### 4.2. Woven Polymer Polylacticacid (PLA) Composites

Baghai et al. [79] studied the effect of the fabric weaving structure on the mechanical behaviour and moisture absorption of the PLA/hemp woven fabric as a reinforcement for the manufacture of composites in the structural component application. The unidirectional woven fabric composites was made from PLA and PLA/hemp wrapped-spun yarns with two distinct weaving patterns: satin and basket. The findings revealed that the composite made of satin-weave architectural fabric had the highest tensile, flexural, and effect strengths compared to the composites made of basket-weave fabric along with a decline in void content and fibre misalignment. Thus, for structural component applications, the satin-weave architecture fabric is appropriate.

**Figure 15:**
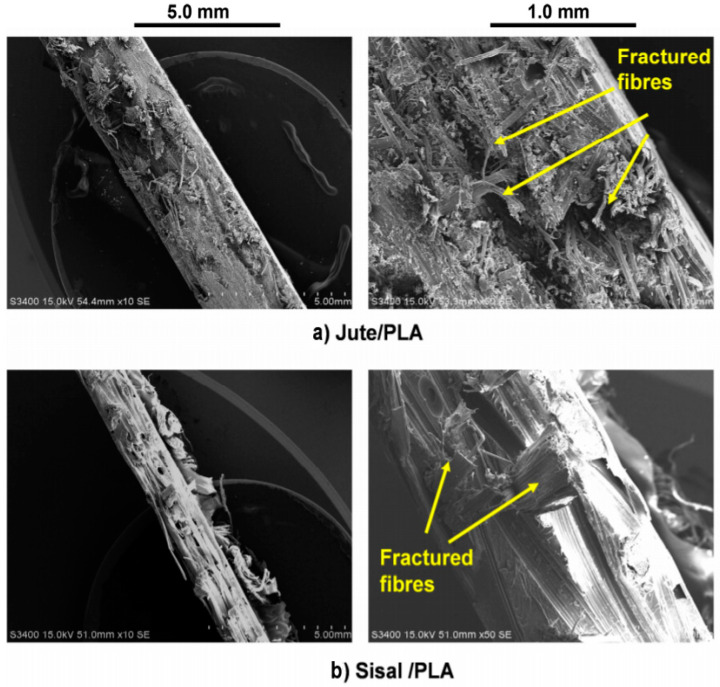
SEM images of the fractured surface of tensile tested specimens at two different magnifications of (**a**) jute/PLA, and (**b**) sisal/PLA. Source: Figure republished from Kandola et al. [87] with permission from Elsevier.

Kandola [87] examined the mechanical and fire properties of hybrid composites with the impacts of natural fibre-reinforced thermoplastic composites from woven-nonwoven. In order to strengthen polylactic acid (PLA) matrices, natural fibre reinforced composites were produced using woven jute and sisal fabrics. The PLA composites were also less flammable and produced less smoke compared to the neat PLA composites. The reinforcement of woven jute and sisal fibres had increased the mechanical properties of hybrid composites. Figure 15a,b show that the holes were formed by the fibre pull out during the tensile testing. This can be explained by changes between the chemical structures of each component. Jute and sisal are cellulose fibres containing the hydroxyl group on the surface, therefore their compatibility with non-polar polymers such as PLA is poor. Therefore, in order to overcome this problem, natural fibre needs to undergo some treatments [157].

A number of research works have been carried out on the production of biocomposites to provide sustainable packaging by using bamboo fibre as a reinforcement agent in PLA composites (bamboo fabric-reinforced composites). One of the studies was carried out by Fazita et al. [158] to determine the bamboo fabric strengthened poly(lactic) acid (BF-PLA) recyclability and biodegradability of composites. The BF-PLA composite was recycled by the process of granulation, extrusion, pelletisation and injection. The mechanical and thermal properties of recycled BF-PLA composites were established in their study and compared with BF-PLA composites and virgin PLA. The mechanical (tensile, flexural, and impact strengths of the recycled BF-PLA (80 MPa, 143 MPa, and 103 J/m) composite were higher relative to the recycled BF-PLA (74 MPa, 156 MPa, and 71 J/m) and neat PLA (61 MPa, 105 MPa, and 37 MPa respectively). The determination of the biodegradability of BF-PLA composites revealed that the biodegradation of the PLA matrix was potentially dependent on the presence of the bamboo fabric and the composting conditions. The incorporation of the bamboo fabric had enhanced the microorganisms’ affinity to the composites under real composting conditions. The biodegradation half-life of BF-PLA composites (46 days) was found to be longer than that of virgin PLA (25 days), indicating that the rapid microbial decay of BF-PLA composites was minimised by the reinforcement of bamboo fabric in the PLA matrix. With the addition of bamboo fabric in the virgin PLA from 151.70–154.42 °C, where the melting temperature for the recycled BF-PLA was found to be 154.56 °C, the melting temperature was found to rise slightly in terms of thermal properties. The process of recycling of BF-PLA composites did not affect the melting temperature of the composites.

**Figure 16:**
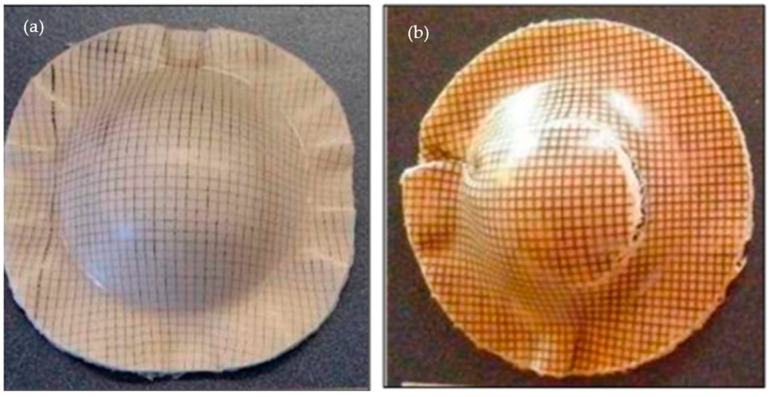
Bamboo fabric-PLA composite laminates with different fabric layups formed using (**a**) hot tooling and (**b**) cold tooling conditions. Source: Figure reproduced with copyright permission from Fazita et al. [159]. This article is an open-access article distributed under the terms and conditions of the Creative Commons Attribution (CC BY) license (http://creativecommons.org/licenses/by/4.0/).

Fazita et al. [159] then continued the continuity of the study on bamboo fabric-reinforced PLA composite. The objective of the experiment discussed was to investigate the influence of various parameters on the incidence of deformations during sheet forming with bamboo fabric-PLA composites of double curvature shapes. The results showed that domes formed under hot tooling conditions were better in quality than domes formed under cold tooling conditions, as can be seen in Figure 16. Wrinkles were deeper in the warp direction of the composite domes, particularly in comparison to the weft direction.

### 4.3. Woven Polymer Epoxy Composites

Sapuan and Maleque [160] explained the appropriateness of the design and fabrication of woven banana fabric-reinforced epoxy composites for household telephone stand manufacturing applications. The telephone stand with woven banana and epoxy is exclusive and aesthetically satisfying in appearance. The telephone stand colour, that is golden brown, is advantageous, because it can be easily fitted with the typical furniture colour. They noticed that banana fibre has the potential to be used in household furniture applications as a reinforcing material in the production of reinforced composites, thus replacing the use of traditional metallic and non-metallic materials. The mechanical characteristics of woven banana fibre strengthened by epoxy composites for household utilities was also studied by Sapuan et al. [161]. The performance composites of three different samples were evaluated and composites were found to be very mechanically stable.

In particular, woven hemp fibre composite was studied by many researchers in the use of hemp fibre for industrial applications. De Vasconcellos et al. [162] studied the resistance to low-speed effects of woven hemp/epoxy matrix composites and the results showed that this composite had an impact behaviour comparable to other synthetic composites. Song et al. [76], on the other hand, investigated the physical behaviour of composites of hemp/poly (lactic acid) (PLA), in particular, the thermal properties and viscoelastic behaviour in the manufacturing of composite parts for automotive and aerospace components. As reinforcements and hemp fabrics-reinforced PLA composites were created, twill and plain woven hemp fabrics were used in this study. The thermal and viscoelastic behaviours of composites of hemp/PLA, which are important for long-term dimensional stability and durability, were studied. Due to the structural features of the twill fabrics, such as less interlacing and tighter packing, the composites strengthened by twill woven hemp fabrics showed stronger mechanical, thermal, and viscoelastic behaviours than those strengthened by plain-woven hemp fabrics. The thermal properties of hemp/PLA composites decreased dramatically with an increase in the volume fraction of fibre, suggesting that composites have a high capacity for parts with a broad range of temperatures, such as automotive and aerospace applications.

Abdellaoui et al. [82] established the use of jute fabric as reinforcement in the thermosetting matrix for application of reinforcing components, i.e., in construction and furniture. They studied the mechanical characteristics of laminated composites based on woven jute and epoxy resins that affected the mechanical behaviour of composites through the effects of numerous layers and fibre directions. The results showed that the laminated composite mechanical behaviour was enhanced by increasing the number of layers and composites in 0° fibre directions.

In ballistic laminate composites applications, the use of woven natural fibre reinforced composites was established. Yahaya et al. [81] investigated the woven kenaf-Kevlar hybrid composites with the effect of layering series, chemical treatment with NaOH, and woven kenaf material. It was stated that the tensile properties of hybrid composites was increased in 3-layer composites compared to 4-layer composites. Hybrid composite with Kevlar as the outer layers demonstrated stronger mechanical properties. Tensile and flexural properties of treated hybrid composites were stronger than untreated hybrid composites. In the case of woven kenaf content, with an increase in kenaf content, the ballistic properties of hybrid composites were reduced. They also found that the arrangement of composite panels had a major impact on the ballistic performance of hybrid composites, whereas, thick composites with double woven kenaf layers performed better in terms of ballistic properties. Furthermore, Rashid et al. [74] explored the potential of coconut coir as a potential reinforcing material for high impact resistance, such as body armours due to its ability to resist high-speed impact penetration.

Woven natural fibre (silk, cotton, lyocel and bamboo)-reinforced composite materials for medical imaging were developed by Morris et al. [32]. Via tensile testing, a three-point flexural modulus bend and clinical imaging using MRI and X-ray, natural fibre composites were shown to provide a promising alternative to ubiquitous carbon fibre skin in return for a small reduction in flexural modulus. Unmercerized cotton or bamboo-based fibres infused with an epoxy or UV matrix were determined to be the optimal materials, offering minimal visibility on all modalities and a flexural modulus at 71% that of carbon fibre. According to their appropriateness for use in MRI and X-ray applications with minimal impact on the resulting images, such materials will enable the production of multimodal composite patient positioning devices. This addresses the increasing need for a truly multimodal patient positioning and support system in the planning of radiotherapy treatment.

In addition, Balasubramanian et al. [151] recently conducted experiments on the effect of sequential positioning of fibre mats on the enhancement of natural fibre (sisal, aloe vera, and flax) hybrid composites properties. An investigation was conducted into the influence of the positioning of fibre mats in bio hybrid composites. S1 (with arrangement of aloe vera mat—flax mat—sisal fibre—flax mat—aloe vera mat) and S2 (with arrangement of flax mat—aloe vera mat—sisal fibre—aloe vera mat—flax mat) were fabricated with epoxy resin as a binder between the fibre mat layers. The study showed that S2 exhibited improved tensile strength, flexural strength, impact strength, and hardness properties. A crucial recognition of the outcome of the experiment was that the choice of fibre mat for outermost or peripheral positioning played an important role in improving the composites’ mechanical properties. This research showed that the hybrid composite formed with flax fibre mat placed at the peripheral layers (S2) had higher tensile strength, flexural, impact, and hardness strength than the hybrid composite formed with aloe vera fibre mat at the peripheral layers (S1). This research also demonstrated that the flexural and impact properties of epoxy reinforced biofibre mats were moderately superior to those of neat epoxy material. In addition, the water-absorbing properties of these reinforced biofibres allowed the composite to be used for structural components used in humidity prevailing environments. It can therefore be inferred that in the case of laminated hybrid composites, apart from a careful selection of biofibre mats, the placement of the fibre mats played a key role in determining the strength and efficiency of the composite material.

The effect of barium sulphate on mechanical, DMA, and thermal behaviour of woven aloe vera/flax hybrid composites were investigated by Arulmurugan [150]. The HNRP5 composite had a maximum tensile strength of 34.72 MPa, mainly based on the interlocking of the flax fibre and the plasticity of the composite was improved by the influence of BaSO_4_. The impact strength decreased significantly with the addition of barium sulphate in the composite. A weight reduction of about 7–9% was recorded in the 100–200 °C temperature range. Monocomposites (HNRP1&2) absorbed 4.8 and 3.5% of humidity, respectively, with the addition of BaSO_4_, the same combination absorbed 4.2 and 3.2% of water content, due to the low water absorption capacity of BaSO_4_. The storage module of the HNRP5 composite had a maximum size in the glass region and a minimum size in the rubber region.

Al Hajaj et al. [163] carried out the impact characteristics of a new hybrid composite material, made of woven carbon fibres plus flax fibres in the epoxy matrix. The purpose of this study was to identify the impact properties of a new hybrid composite made of woven carbon fibres plus unidirectional (Type A) or cross-ply (Type B) flax fibres in an epoxy matrix. A pendulum impact tester used photographic, thermographic, and geometric techniques to evaluate a range of impact energies (5–40 J) on a series of composite plates. As noted by lower absorbed energies, higher penetration energy, smaller crack lengths, smaller indentation depths, smaller damage areas, lower temperature increases in the impact zone than on applied impact energy, and greater impact strength, the Type B composite had better impact performance than on Type A. Compared to pure flax fibre-reinforced epoxy composites noted in earlier literature, both Type A and B hybrid composites had superior impact properties, proposing that hybridisation can be done effectively using synthetic and natural fibres.

Mode-I interlaminar fracture strength of flax, glass, and hybrid flax-glass fibre woven composites was investigated using the DCB test of Saidane et al. [90]. To examine the damage mechanism of each composite, the acoustic emission signals obtained during the tests and scanning electron microscope images were used. The crack initiation requires the highest energy for the flax-fibre laminate (1079 vs. 945 for hybrid flax-glass fibre and 923 J/m^2^ for glass fibre laminates). As the origin of this highest energy, the morphology of the flax fibres, short and bonded together in bundles to produce the twill fabric, allowed the creation of a greater quantity of fibre bridging. In addition, the hybridisation of glass fibres with flax fibres in an acceptable combination provided a fascinating alternative when it was required to increase the strength of glass fibre composites. The significant gain of the weight reduction of the composite structure was due to the low density of flax fibre is more interesting.

Chee et al. [91] produced a non-woven bamboo mat hybridised with a woven kenaf mat and reinforced by hand lay-up method in an epoxy matrix to establish a cost-effective high-performance hybrid composite for automotive and construction applications, where great concerns were given to dimensional stability and mechanical properties with temperature variation. The total loading of the fibres in this study was controlled by 40% by weight, the fibres mat were arranged in a layer by layer sequence. The results of the review showed that the woven kenaf mat improved the dimensional stability of the composites. B:K:50:50 hybrid composites showed the best dimensional stability of thermomechanical and dynamic mechanical properties. Bamboo/epoxy composite complex and storage modules were higher compared to kenaf/epoxy composites. Complex and storage modules of the composites followed the sequence series of bamboo/epoxy > B:K:50:50 > B:K:70:30 > B:K:30:70 > kenaf/epoxy > pure epoxy. B:K:50:50 hybrid composite showed the lowest value of the efficiency coefficient and the lowest damping behaviour composite which implied the effective stress transfer and the highest interfacial adhesion strength of all composites.

Palani Kumar et al. [164] have conducted a study on the mechanical properties of aloe vera/sisal/kenaf fibre reinforced epoxy composites. The composite laminates were manufactured using a hand lay-up technique. Tensile, flexural and impact tests were performed for woven aloe vera and kenaf (AK), sisal and kenaf (SK), aloe vera, sisal, and kenaf fibre hybrid epoxy composites (ASK). From the experiment carried out, the SK hybrid composite showed good tensile properties by giving tensile stress of 28.59 MPa. In the meantime, AK hybrid composite exhibited improved flexural properties by up to a maximum of 1.59 MPa. The greatest impact strength for ASK hybrid composite with a value of 6.20 J was noted due to the integration of three different fibres into the composite.

### 4.4. Woven Polymer Polyester (PE) Composites

Alavudeen et al. [78] established banana/kenaf fibre-reinforced hybrid polyester composite for structural applications using two distinct weave architectures, which included plain and twill, with particular reference to treatments of resin fibre, weave architecture, bonding properties, and modification of fibre surface. In this analysis, the mechanical strength of woven banana/kenaf fibre hybrid composites were increased due to the hybridisation of kenaf with banana fibre. The tensile, flexural and impact strengths of the woven hybrid composite of banana/kenaf fibre were higher than those of the individual fibres. Of the two weaving patterns, the flat form showed improved tensile properties compared to the twill type in all the fabricated composites, suggesting stronger fibre-matrix adhesion in the hybrid composite due to the interlocking of the woven fibre.

Pothan et al. [165] produced woven banana-glass-fibre reinforced composites for construction materials. The composites were prepared by impregnating the woven banana fibre in preform (fabric) with polyester resin. They indicated that better tensile strength can be achieved by using two layers of fabric due to the increase in the number of layers that made the second relaxation peak noticeable and the damping values found to be reduced by the addition of more layers. They also concluded that the treatment of banana fibre with silane and sodium hydroxide (NaOH) resulted in a maximum increase in modulus values, as the chemical adjustment enhanced the storage modulus of banana fibre reinforced polyester composites. This composite of woven banana and glass fibre and polyester resins is suitable as a replacement for building material due to better modulus and low damping properties.

For the thermoplastic matrix, Ahmed et al. [71] investigated the low-speed tolerance impacts of the woven jute/polyester composite. Composites were checked with 100% jute fabric and hybrid composites, made of jute fabric with the addition of glass fabric plies. They demonstrated that the jute-reinforced composites had a higher potential for absorbing impact energy but a lower tolerance for damage than the hybrid glass/jute composites.

In components such as false ceilings, floor and ceiling tiles, columns, windows, structural insulated panels, desert structures, low-cost houses, and other structural uses, Dalbehera and Acharya [166] recommended the usage of woven jute glass-reinforced in composites. In this study, it was found that woven jute and glass of reinforced fabric composites filled with ceramic rich cenosphere (as filler) had a significant impact on erosion behaviour. The filler content was identified as the most important factor affecting erosion wear and improves erosion resistance.

A study on the performance of mechanical properties of woven kenaf-glass reinforced polyester composites for the structural application was conducted by Atiqah et al. [167]. Flexural strength of the kenaf-glass hybrid composite for the treated 15% composition showed higher values of flexural, tensile, and impact strength compared to other formulations (12.5% and 22.5%). The mercerization of the 15% volume fraction treated kenaf showed the highest tensile modulus, which increased the rigidity of the hybrid composite. The kenaf fibre alone cannot withstand higher impact loads leading to brittleness and less strength in hybrid composites. Mercerization was shown to enhance the adhesion between the fibre surface and the matrix, which played a key role in enhancing the mechanical properties of hybrid composites. The adhesion between the fibre and the polyester matrix were affected by the existing properties of each fibre.

Hamdan et al. [168] examine the impact of water absorption on the various types of natural woven fibre (i.e., jute, ramie, and roselle) reinforced with polyester resin. The study of water absorption and thickness swelling was performed with a composite sample immersed in distilled water for 30 days. In their major study, they found that the result of the tensile properties showed that the layering size had more influence than the layering sequences. The tensile strength value of the two-layer composite was approximately 26.22–29.18 MPa, while that of the three-layer composite was 28.18–32.90 MPa. The hybrid of the ramie-jute-ramie was larger with a tensile value of 32.90 MPa. The jute-roselle-jute hybrids, meanwhile had both higher tensile strength and impact strength, with values of 49.50 MPa and 75.4 KJ/m^2^, respectively. Based on the results obtained, a single form of water absorption and a hybrid composite sample yielded approximately 3–6%. In addition, the effect of the swelling thickness was at a minimum, particularly in the initial phase of immersion (1–7 days). Absorption of water has a detrimental effect on tensile properties. The tensile strength and the tensile modulus experienced a great loss in strength and flexibility when submerged in distilled water. Void content affected the efficiency of the tensile properties. The absorption of water decreased the tensile strength on the 30th day by about 12–27% and the tensile modulus by 15–35%. The result showed that the resistance to water absorption towards hybridisation had improved significantly.

Ahmed et al. [169] provided a significant study and discussion on the impact of stacking sequences on tensile, flexural, and interlaminar shear properties of untreated woven jute and glass fabric reinforced polyester hybrid composites. The laminates were manufactured by a hand lay-up technique in the mould and cured under light pressure for 1 h, followed by curing at room temperature for 48 h. All laminates were made with a total of 10 plies, depending on the number and location of the glass layers, in order to achieve six separate stacking sequences. In a study conducted by Ahmed et al. [169], it was shown that the properties of jute composites can be significantly improved by the addition of glass fibre as extreme glass plies. The layer sequence is more efficient than tensile properties for flexural and interlaminar shearing properties. The overall contrast between the properties of all laminates showed that the hybrid laminate with two extreme glass plies on each side was the optimum mix with a strong balance between properties and costs. Incorporation of glass in jute fibre composites increased the tensile strength, flexural strength, and interlaminar shear strength of the resulting hybrid composites, with values of 125 MPa, 160 MPa, and 16.5 MPa, respectively. The flexural and interlaminar shear strength was significantly influenced by the layering sequence (altering the position of glass plies). The layering sequence has little effect on tensile characteristics for the same relative weight fraction of jute and glass fibre. The overall contrast between the properties of all the laminates showed that the hybrid laminate (S3) with two intense glass plies on each side was the best mix with a strong balance between the properties and the expense (which increased with the rise in the amount of layers of glass).

In addition, a research study conducted by Ahmed et al. [170] identified the effect of hybridisation on the mechanical properties of untreated woven jute and jute-glass isothalic polyester composites. They discovered that tensile properties, such as tensile strength and tensile modulus and the impact properties of jute-polyester composites were improved by the addition of glass fibres with a positive hybrid effect. The addition of 16 wt% of glass fibre resulted in the highest flexural and interlaminar shear strength (ILSS). Adding 16.5 wt% of glass fibre to a total fibre weight fraction of 42% raised tensile, flexural, and interlaminar shear strength (ILSS) by 37, 31.23, and 17.6%, respectively. The addition of glass did not demonstrate any changes in these properties, even though an increase in flexural modulus was noted. Fractography of fractured surfaces of jute composites revealed poor adhesion of jute fabric to the matrix. As a result, an effort can be required to enhance the adhesion by appropriate surface treatment of the jute fabric. The specific impact strength of hybrid laminate (IH3) with a glass-weight fraction of 17.2% was comparable to the specific impact strength of glass-polyester composite with a 60% fibre-volume fraction.

Khalid et al. [171] investigated the woven composite structure of twill yarn kenaf. Composites were prepared in this experiment using the hand lay-up method with different orientation types, where the orientation was designed using the Taguchi method. The hardened composites were treated at ambient temperature for 24 h before forming in accordance with ASTM D3039. The samples were then emphasised uniaxially in order to obtain the stress-strain curves. The result showed that fibre orientation was a significant factor in the determination of tensile strength efficiency. In this work, the fibre mats were then optimised and the results indicated that the tensile and modulus strength values were 55.738 MPa and 5761.704 Joule, which increased for 3.77% and 4.23%, respectively for tensile strength and Young modulus. By contrasting the fracture mechanism before and after optimisation, the fracture surface was decreased significantly. It was noted that by this method, the mechanical behaviour performance of the twill woven kenaf reinforced composites could be improved effectively.

In a study to evaluate the effect of organo-modified nanoclay (OMMT) on the mechanical properties of sugar palm fibre reinforced non-saturated polyester (UP) composites, Shahroze et al. [172] found that the addition of OMMT resulted in substantial improvements in all mechanical properties up to certain loading times. OMMT (1–5%) and sugar palm fibre were introduced in UP and mixed using a mechanical stirrer and then hot compressed to form the composites. The result showed that the 2% and 4% nanoclay content showed optimum tensile and flexural and impact properties, respectively. The inclusion of nano-reinforcement in resin resulted in the increase in the usability of these sites for efficient bonding and improved transferability of stress. The acquisition of these sites on a nanoscale gave rise to a higher net fibre-matrix contact area, which led to a relatively higher rate of strength improvement.

## 5. Applications of Woven Natural Fibre Polymer Composites

High strength fibres like Kevlar, carbon fibre, fibre-glass, Aramid and carbon nanotubes are commonly used in advanced composite structures as composite reinforcing materials. Nevertheless, the world is changing and green materials are in the vanguard due to the depletion and health concerns about inorganic materials, such as petroleum. In addition, the growing demand for natural fibre for composites has increased rapidly due to cost-effectiveness, low density, abundant, good thermal and insulating properties, renewability, biodegradability, high specific strength, etc. [173,174].

These successful benefits have stimulated the interest of numerous researchers in recent years in the development of natural fibres as reinforcement in polymer matrix composites such as kenaf fibre [89,175,176], oil palm fibre [177,178], sugar palm fibre [179,180], banana fibre [181,182], pineapple leaf fibre [183,184], flax [185,186], hemp [187,188], sisal [189,190], coir [191,192], jute [193,194], etc, as summarised in Table 4.

The development of loose natural fibre into a woven form improved the natural fibre’s potential and usefulness as a reinforcement in the advanced composite structure. In recent years, an increasing interest in textile composites has been observed. They are progressively used in the fabrication of high mechanical performance structures in many fields as listed in Table 5.

The woven-fibre composite material denotes the type of textile composite in which strands form through weaving process; they are interlaced with each other and impregnated with a resin material in two mutually orthogonal (warp and fill) directions. Particularly, in the form of short and random, composite materials reinforced with woven fabric have better out-of-plane rigidity, strength, and durability properties than laminate composites, although the geometry of this composite class is complex and there is no limit to the choice of possible architectures and components. Many parameters of woven-fibre composite materials can be altered, such as the geometry of microstructures, weave type, and hybridisation or choice of components (e.g., geometric and mechanical parameters of strands and resins) [223]. Thus, their mechanical performance should be taken account of in the preliminary study for the selection of woven fibre composites with the right blend of weight, cost, toughness, and strength properties.

Biocomposites must have useful characteristics (high-quality performance, durability, and reliability standards) in order to replace synthetic fibre-reinforced composites and extend into other industrial applications, such as traditional petroleum plastics used in automotive applications in particular [4,5,25,224]. The quest for lightweight vehicle parts, together with good end-of-life disposal, has indirectly opened a gateway to solve the issue of fuel consumption in the automotive sector and thus, reduced greenhouse gas emissions [225]. With this in mind, the European Commission has implemented the “European Guideline 2000/53/EG” which set the objective of improving the vehicle’s recyclability to 85% by weight in 2005. This percentage was increased to 95% by 2015 [225].

Longest fibres, such as jute, can be formed into flexible fibre mats that can be produced by physical entanglement, nonwoven needling, or thermoplastic fibre melt matrix technologies. The two major types are carded and needle-punched mats. In carding, the fibres are combed, mixed and physically entangled in a felted mat. Geotextiles have a wide range of applications. It can be used as a mulch around the freshly planted seedling. With good moisture retention and promoting seed germination of jute fibre mats, low-and medium-density fibre mats can be made and used for soil stabilisation around new or existing constructions to stop soil slopes without roots from soil erosion and topsoil loss. As natural separators between various materials, medium and high-density fibre mats can also be used underground in road building and other forms of construction. Woven jute is commonly used as a “gunny” and tote bag because of the long and strong properties of jute fibre.

Morris et al. [32] investigated woven natural fibre-reinforced composites materials for medical imagery. The various woven natural fibre materials, such as silk, cotton, lyocel, bamboo, and carbon fibre (as control), were integrated into a range of various resin materials appropriate for such applications. From examining a variety of resins and natural fibre materials in combination and testing their performance in terms of MRI and X-Ray imaging, the result showed that a woven cotton material impregnated with a two-part epoxy resin increased the passage of X-Rays by 15% and had no effect on the MRI signal (unlike the 40% MRI signal attenuation from carbon fibre) while maintaining a flexural modulus up to 71% of that of carbon fibre. These findings showed that natural fibre composites generated using such materials have attractive properties for use in patient care and positioning devices for multi-modal imaging without the need to substantially compromise the strength of the material.

In biomedical applications, Bagheri et al. [226] suggested flax sandwiched with thin carbon sheets on either side for use as an orthopaedic long bone fracture plate because the hybrid’s mechanical properties are closer to the human cortical bone than the clinically used orthopaedic metal plates, rendering the material a possible candidate for long bone fracture fixation. A sandwich-structured composite in which two thin sheets of carbon fibre/epoxy are attached to each outer surface of the flax/epoxy core, resulting in a unique structure compared to other bone plate composite plates. The findings of the mechanical testing showed a significantly high final strength in both tension (399.8 MPa) and flexural loading (510.6 MPa) with a higher elastic modulus in bending tests (57.4 GPa) relative to tension tests (41.7 GPa). In both tension and bending tests, the composite material suffered a brittle catastrophic failure. Compared to clinically use orthopaedic metal plates, current CF/flax/epoxy findings were similar to human cortical bone, making the material a possible candidate for long bone fracture fixation.

In sporting good uses, a multitude of flax preforms have been used: woven flax prepregs, containing epoxy resin, were combined with carbon prepregs in the hybrid material concepts used in tennis rackets, bicycles, fishing rods, or ski. Glass fibres are still used in the latter and in one particular technology [227], the core of the ski sandwich structure is reinforced with strips ± 45° flax, located in the thickness direction of the core (balsawood), facilitating more weight reduction and damping capability improvement. In ski poles, braided flax is used, making them a bio-based and light alternative to poles reinforced with carbon or glass fibre.

Wambua et al. [228] found that flax composites had higher energy absorption compared with hemp and jute composites. However, the ballistic qualities of hemp composites were improved greatly when a mild steel plate was used to protect and support the body armour. Radif et al. [229] noticed that the use of a woven ramie Kevlar-reinforced polyester composite as a material to manufacture body armour, in particular, to reduce the amount of Kevlar used, created a potential cost-effective product and could also contribute to a reduction in its cost of production. Furthermore, the armour was comparable to the third level of protection of the ballistic limits in accordance with the International Standard of the National Institute of Justice (NIJ).

A multilayer armour system (MAS) in which traditional Kevlar^TM^ was substituted by either an epoxy matrix composite reinforced with 30 vol% jute fabric or a plain epoxy plate following an NIJ trauma limit after ballistic testing with 7.62 mm ammunition was manufactured and characterised by Luz et al. [230]. Within the statistical deviation, the ballistic output for the three investigated MAS second layer materials were found to be identical. Generally, lesser energy was dissipated by the aramid fabric in perforating individual ballistic tests, while the jute fabric composite and the plain epoxy were more effective. While not indicative of what happened in the MAS tests, the energy dissipated by each particular substance led to the exploration of the ballistic significance of the epoxy rupture mechanism. Referring to them, evidence of the massive collection of post-impact fragments by the composite of jute fabric and also the aramid fabric, which has also been recently reported, has been suggested as the main mechanism for energy dissipation. Additional fragment capture and brittle epoxy spalling (plain or composite matrix) contributed significantly to the dissipation of post-impact energy. Despite comparable ballistic efficiency and a negligible difference in weight, the considerably lower cost associated with environmental and societal advantages of natural fibre in practice favoured the replacement of jute fibre composite for both aramid and plain epoxy in MAS.

Woven bamboo fabric is similar to silk in softness. The fibres, which are not chemically treated, are generally smoother and more round without sharp spurs to irritate the skin making bamboo fabric hypoallergenic and ideal for people who have allergic reactions to other natural fibres like wool or hemp. A comparative analysis by Tausif et al. [231] on their comparative study of bamboo viscose fibre as an eco-friendly alternative to cotton fibre in knitted polyester-cellulosic blends was performed. Conventional cotton is not known to be eco-friendly, since it needs a significant quantity of water and pesticides during its processing. The eco-friendly quality of bamboo viscose is subjected to the manufacturing process used. Polyester-bamboo (PB) and polyester-cotton (PC) blended yarns were prepared using open-ended spinning techniques and the yielded yarns were single jersey weft-knitted. The results indicated that the PB blend outperformed the PC blend in mechanical properties and demonstrated lower thermal resistance than the PC blend, which is advantageous for summer clothing. Although at higher proportions of bamboo viscose fibre in the PB blend, the moisture management characteristics of PB blended fabrics were expected to be comparable to those of PC blended fabrics.

## 6. Challenges and Future Perspective on Woven Natural Fibre Composites

Under the Industrial Revolution 4.0 (IR4.0), development of woven natural fibre composites remains in very high challenge. The woven polymer composites are normally fabricated with thermoset matrices, although thermoset composites are well-known for their superior strength [86]. However, the fabrication processes in innovative ways are hardly ever reported [232]. Additives manufacturing (AM) coves the 3D printing production from a thermoplastic polymer, ceramic to metal materials. It reduces waste materials, labour monitoring and energy used [233]. The insertion of natural fibre reinforcements in powders and yarn forms, into PLA polymer composites, were reported as FDM or SLS 3D printing materials [234]. Unfortunately, there is no room is available for woven natural fibres composites in the additives manufacturing, at least not to date. As a future perspective, woven natural fibres should be involved in AM to produce strong composites at a minimum cost.

Despite the intense research conducted on woven natural fibre composites, the use of woven natural fibre in aircraft interior components is not yet seen. Aircraft components have strict regulations on their durability and performance, especially with regards to flame retardancy [235]. However, woven natural fibres behave similarly as wood materials, with high flammability and low thermal stability in general [236]. Although numerous treatments and flame-retardant fillers were included in the woven natural fibre composites’ design, and enhanced thermal and flame properties were achieved, but compliance with aviation materials’ requirements seems still challenging. Therefore, pushing the woven natural fibre composites as the alternative aircraft interior materials would be prior task needed to be done instead of continuous research without commercialisation.

As commercialization is a part of the future schedule, the popularisation of woven natural fibre composites is important. Without knowing and understanding from the public, commercialising woven NF composite products shall be a tough challenge. Publicity on the woven natural fibre composites should be a collaborative effort etween universities, government and industrial partners. Woven natural fibre composites have been innovative materials among researchers for at least decades, however the public has zero or limited information about these woven natural fibre composite materials. This makes it challenging for companies to use woven natural fibre composites in their products. This is because consumers are not going to select the products with which they are not familiar or confident. Hence, the government should take the initial step to introduce and promote the achievements of woven natural fibre composites conducted by local universities, via social media and newspapers. Through this approach, good products made from woven natural fibre composites can be delivered to consumers.

## 7. Conclusions

Woven composites for high-end engineering applications can be made from natural fibres by matching the quality of the woven fabric. The types of yarn and yarn arrangement in the woven fabric influence the physical and mechanical properties of the resulting woven composite. There are a number of aspects to be considered in the production of woven composites, including yarn types, woven fabric characteristics, manufacturing techniques, and matrix types. Essentially, it is vital to study the structure of yarn forms, linear density, weights, mechanical properties, twist effect, etc., because these factors determine the structure of the woven fabric. For example, example, fabric count, weave design, mobility, etc., will all decide the efficacy of the admixture of the matrix-woven fabric and the composite itself.

## Figures and Tables

**Table 1:** Mechanical properties of different plant fibres.

Fibre	Density	Young’s Modulus (GPa)	Tensile Strength (MPa)	Elongation (%)
Sugar palm	1.5	10.4	421.4	9.8
Bamboo	0.6–1.1	11–17	140–230	-
Cotton	1.5–1.6	5.5–12.6	287–597	7–8
Flax	1.54	27–85	345–2000	1–4
Hemp	1.47	17–70	368–800	1.6
Jute	1.44	10–30	393–773	1.5–1.8
Kenaf	1.2	14–53	240–930	1.6
Ramie	1.5–1.56	27–128	400–1000	1–4

Sources: Sanyang et al. [51]; Ilyas et al. [52,53,54]; Oksman et al. [55]; Bodros and Baley [56]; Ochi [57]; Satyanarayana et al. [58]; Summerscales et al. [59]; Bourmaud [60]; Faruk et al. [61].

**Table 2:** List of reported studies on woven natural fibre reinforced polymer composites.

Fibre Types *	Matrix Type	Key Findings	Ref.
Woven jute: glass fibre (43:0, 35:8, 25:16, 17:25)	Polyester (PE)	Woven jute laminates had poorer damage resistance and tolerance capability than jute/glass hybrid laminates, but better in damage tolerance capability. The optimum ratio of the hybrid laminate was 25:16, that increased the damage tolerance capability.	[71]
Woven flax (31–39%)	Epoxy	The addition of woven flax improved the fracture toughness of the composites and the strength of the fracture depended significantly on the directions (warp or weft) of the composite being tested.	[73]
Plain woven coir and kevlar	Epoxy	Woven hybrid coir/Kevlar composite had flexural strength and impact strength nearest to the properties of woven Kevlar composite.	[74]
Woven kenaf fibre	Glass, nylon fibre	The incorporation of kenaf woven as one of the layers in the existing laminated composite structure in making prosthetic leg sockets resulted in the improvement in the impact, tensile and flexural strength.	[75]
Plain and twill hemp fabric (6%—1 layer and 20% —three layers)	Polylactic acid (PLA)	Composites incorporated with twill hemp fabrics showed better mechanical, thermal, and viscoelastic behaviours than plain hemp fabric composites. Improvement of the twill hemp in the composite was due to the dimensional properties and weave structures of the twill fabrics.	[76]
Kenaf fabric	Epoxy	Yarn size and weave pattern influenced the porosity, unit cell, weft crimp, and warp crimp. The mechanical properties of woven kenaf composite depended on the crimp and porosity.	[77]
Woven banana and kenaf (40%)	Polyester (PE)	Plain fabric improved the tensile properties of the composite compared to twill fabric composite and randomly oriented composites.	[78]
Hemp woven fabric (30%)	Polylactic acid (PLA)	Composite from satin fabric had significantly lowest porosities and better mechanical properties than the composites made from the wound hybrid yarn and basket fabric.	[79]
Ramie fabric (30%)	Epoxy	Ballistic tests revealed that 30 vol% of ramie fabric composites had a better performance than Kevlar as a ceramic front backing plate in multilayered ballistic armour systems with more cost-saving (~95%) of the total multilayered armour.	[80]
Woven kenaf: Kevlar fabric (42–43: 60–62)	Epoxy	Hybrid composite with kenaf fabric as skin layers exhibited higher impact properties compared with samples with Kevlar as skin layers.	[81]
Woven jute (41%)	Epoxy	The mechanical behaviour of woven jute laminated composite was improved by increasing the number of layers, with a maximum number of layers of 5 at 0° fibre and cutting direction.	[82]
Woven kenaf (35%)	Epoxy, unsaturated polyester and vinyl ester.	Plain woven kenaf composite possessed good mechanical strength. Tensile, flexural and impact strengths of the woven kenaf/epoxy composite were superior to those of the other polymers.	[83]
Jute fabric	Poly (L-lactic acid) (PLLA)	Woven jute fabric composites in warp and weft directions presented superior mechanical properties than non-woven jute fabric composites.	[84]
Kenaf fabric: glass fibre (6.8–15.2: 8.8–20.4)	Polypropylene (PP) and epoxy	Fatigue strength of laminated kenaf fabric and glass fibre reinforced with polypropylene demonstrated greater performance compared to composites reinforced with epoxy.	[85]
Woven kenaf: carbon fibre (16–20: 12)	Epoxy	Weave design and fabric count of kenaf fabrics played important roles in determining the final laminated composite properties. The findings showed that plain fabric was more suited to achieving high tensile and impact strengths than satin fabric.	[86]
Plain woven jute (39–42%), plain woven sisal (34–41%) and glass fabrics (72–74%)	Polypropylene (PP) and polylactic acid (PLA)	The PLA composite tensile and flexural modulus were superior to the PP composites. Compared to jute composites, sisal composite has higher tensile and flexural modulus.	[87]
Flax fabrics: carbon fabric (28–31: 8–15)	Epoxy	There were noteworthy improvements in the strength and stiffness of various interlayer flax-carbon hybrid configurations at low carbon volume fractions.	[88]
Kenaf fabric and carbon fibre (16–20%)	Epoxy	Hybrid composite with higher kenaf fibre content (higher fabric count) showed better thermal stability whilst pure carbon fibre composite had the highest thermal stability. The DSC data revealed that the temperature of decomposition increased with the presence of woven kenaf.	[89]
Woven flax: glass fibre (0.26–0.4: 0.14–0.5)	Epoxy	Hybridization of flax fabric in composites with glass fibre showed good resistance to better interlaminar fracture toughness.	[90]
Woven bamboo and kenaf mat (40%)	Epoxy	The optimum weight ratio of hybridization bamboo mat and woven kenaf for good thermomechanical and dynamical mechanical properties of hybrid composites is 50:50.	[91]
Woven kenaf	Epoxy	Adding stitches on the woven kenaf structure gave a better performance in specific strength while stitching patterns and stitching angle gave significant impact to woven stitch kenaf composite performance when compared to unstitched ones.	[92]
Woven jute, ramie and roselle	Polyester	Various types of woven fabric used in the hybrid composite showed various mechanical properties. Better tensile and flexural properties of the composite were provided by the hybridization of woven jute and ramie. In the meantime, hybrid woven jute and roselle developed superior properties of composite impacts.	[93]
Woven aloe vera and flax	Epoxy	The physical properties and woven pattern of the aloe vera fibre improved the toughness properties of the composite. The chemical treatment of aloe vera fibre by using barium sulphate (BaSO_4_) was found to increase the thermal stability of the composite.	[94]
Jute, hemp and flax fabrics (47%)	Polypropylene (PP)	The effect of the fabric architecture, i.e., woven, woven commingled and knitted commingle of jute, hemp, and flax on the composite properties were investigated. The results showed composite from jute knitted commingled fabric composite exhibited 20% and 29% higher strengths compared to their woven and woven commingled composite.	[95]

* Fibre content based on weight.

**Table 3:** Comparison between plain, twill and satin structures.

Properties	Weave Structure		
	Plain	Twill	Satin
Good Stability	****	***	**
Good Drape	**	****	*****
Low Porosity	***	****	*****
Low Crimp	**	***	*****
Balance	****	****	**

Note: ***** = excellent. **** = good, *** = acceptable, ** = poor, * = very poor.

**Table 4:** List of reported study of natural fibre reinforced polymer composites.

Fibre Types	Matrix Type	Properties Remark	Ref.
Oil palm frond	Urea Formaldehyde	Composite with 50% of fibre showed higher flexural strength, modulus of electricity (MOE), and tensile strength of 1.43 MPa, 1248 MPa and 3.8 MPa, respectively.	[195]
Oil palm EFB	Polypropylene	Microwave-treated fibre-based composites showed improved mechanical and thermal properties. EFB fibres treated at 90 °C for 90 min were found to be suitable for better reinforcement into the composite in terms of mechanical, thermal, and crystalline properties. Moreover, onset degradation temperature and water absorption properties were also found to be changed apparently due to treatment.	[196]
Sugar palm	Unsaturated polyester	Increasing trends in the tensile strength, tensile modulus, flexural strength, and flexural modulus were shown in sugar palm yarn loadings of up to 30 wt%. However, maximum impact strength was achieved at 40 wt% of sugar palm fibre yarn loadings. Elongation at break increased with the increment of sugar palm yarn loading up to 50 wt%. The thermal stability of the composite decreased in accordance with onset and maximum temperatures, while the percentage of residue increased for higher fibre loadings	[197]
Sugar palm	Epoxy	Increasing flexural and torsion properties of the non-hybrid composite at fibre loading of 15 wt% were 7.40% and 75.61%, respectively. For hybrid composites, the experimental results revealed the highest flexural and torsion properties that was achieved at the ratio of 85/15 reinforcement and 60/40 for the fibre ratio of hybrid sugar palm yarn/carbon fibre-reinforced composites. The different ratio between matrix and reinforcement had a significant effect on the performance of sugar palm composites.	[198]
Kenaf	Epoxy	The kenaf composite was found to withstand a maximal temperature of 120 °C. The tensile and flexural strengths of the aligned kenaf composites (50 and 90 MPa, respectively) were three times higher than those of the commercialized Product T (between 39 and 30.5 MPa, respectively) at a temperature range of 90 to 120 °C.	[199]
Kenaf	Polylactic acid (PLA)	The acetylation treatment was effective for improving the performance of PLA/kenaf composites. This behaviour was found to relate to the surface cleaning of acetylated kanaf, in addition to the efficient modification of the hydrophilic characteristics of kenaf.	[200]
Banana fibre	Epoxy	The mechanical analysis indicated that 6% NaOH treatment with a two-hour immersion time gave the highest tensile strength. It was found that 6% NaOH treatment with a two-hour immersion yielded the highest interfacial shear stress of 3.96 MPa. The TGA analysis implied that alkaline treatment improved the thermal and heat resistivity of the fibre.	[201]
Banana fibre	Epoxy	The best mechanical performance was achieved in the composite specimen of 10 mm fibre length and 15% fibre loading.	[202]
PALF	Epoxy	The continuous and aligned fibres significantly increased flexural strength. Revealed by Weibull statistics, the PALF reinforcement, above 10–30 vol%, followed a linear increase to a value of flexural strength around 120 MPa.	[203]
PALF	Epoxy	It was found that the change in fibre orientations will have a great influence on storage modulus and loss tangent along with other mechanical properties investigated in this study, as the evidence from the table that maximum variance in storage modulus at frequencies of 0.1, 1 and 10 Hz were approximately 3.86, 4.26 and 4.23 GPa, respectively and the corresponding variance in loss factor values were 0.16, 0.12, and 0.09, respectively.	[204]
Flax	Epoxy	The unstitched and stitched flax composites showed that while delamination was not the predominant damage mode in both laminates, stitching did facilitate the propagation of in-plane cracks. The findings revealed that stitching with thicker yarn (flax) led to a lower ratio of absorbed energy per area of damage as well as the energy absorbed for full penetration.	[205]
Flax	Vinyl ester	The results at two different impact energies (25 J and 50 J) confirmed the fact that the flax specimens can absorb more energy during the impact event, but they tended to show greater damage extension at lower energy levels compared to the glass/flax specimens. The hybridisation of the flax reinforced natural fibre composite revealed a much higher impact performance, exhibiting greater perforation and penetration resistance with the benefits of having a lower environmental impact than glass fibre laminates without hybridisation.	[206]
Hemp	Polyurethane (PU)	Increasing fibre volume content to 40%, flexural strength enhanced 193.24%. Additionally, 15 mm hemp fibre was found to be the optimum fibre length, where flexural strength at 40% fibre volume was further increased to 274.3%.	[207]
Hemp	Unsaturated polyester	The results suggested a significant effect of chemical treatment in terms of increasing mechanical and dynamic mechanical properties and decreasing in water absorption properties. The benzoylation treatment showed a better impact among all three chemical treatments (benzoylation, alkali treatment, and sodium bicarbonate).	[208]
Sisal	Epoxy	The results indicated that storage modulus and loss modulus were found to be high for the composite having 15 mm length of fibres.	[209]
Jute	Epoxy	Composites prepared from chemically treated (acid pretreatment, alkali pretreatment, and scouring) jute fibres were found to be better than the raw jute composites in terms of tensile strength, elongation at break, void fraction, and interfacial adhesion. The findings suggested the chemical treatment of jute fibres could enable better matrix–fibre adhesion due to improvement in interfacial bonding with polymer matrix, which consequently improved the tensile properties of the composites.	[210]

**Table 5:** Applications of woven natural fibres composites in field scales.

Fibres	Reinforcement	Applications	Ref.
Bamboo fabric	Polylactic acid	Packaging	[158,159]
Woven jute and glass fibre	Polyester	Solar parabolic trough collector	[211]
Twill weave woven flax fabric	Polypropylene	Marine composite	[212]
Woven kenaf bast and oil palm EFB	Polyhydroxybutyrate (PHB)	Construction and building materials	[213]
Flax fabric	Carbon nanotubes	Supercapacitor electrode	[214]
Sisal fabric, flax fabric and glass fibre	Epoxy	Wind turbine blades	[215]
Woven cotton fabric	Polylactic acid	Antibiotic delivery device	[216]
Plain hemp fabric	Epoxy	Electronic racks	[217]
Woven kenaf and Kevlar fabric	Epoxy	Ballistic armour materials	[27,81]
Sugar palm yarn	Polyester	Automotive component	[198,218,219,220,221,222]

## Data Availability

Not applicable.
